# Dietary Omega‐3 Long‐Chain Polyunsaturated Fatty Acids Can Enhance Ecologically Relevant Cognitive Traits in Juvenile Brown Trout

**DOI:** 10.1002/ece3.72340

**Published:** 2025-10-16

**Authors:** Stefano Mari, Stefan Auer, Benedikte Austad, Pernilla Hansson, Simon Vitecek, Mourine J. Yegon, Libor Závorka

**Affiliations:** ^1^ Department of Functional and Evolutionary Ecology University of Vienna Vienna Austria; ^2^ WasserCluster Lunz—Biologische Station, Inter‐University Center for Aquatic Ecosystem Research Dr. Carl Kuperlwieser‐Promenade 5 Lunz Am See Austria; ^3^ Department of Water, Atmosphere and Environment, Institute of Hydrobiology and Aquatic Ecosystem Management University of Natural Resources and Life Sciences Vienna Austria; ^4^ Department of Biological & Environmental Sciences University of Gothenburg Gothenburg Sweden; ^5^ Department of Ecology University of Innsbruck Innsbruck Austria

**Keywords:** behavioural types, Brown trout, cognition, Omega‐3 fatty acids, resource acquisition

## Abstract

Laboratory experiments have demonstrated that dietary intake of omega‐3 long‐chain polyunsaturated fatty acids (n‐3 LC‐PUFA) is beneficial for survival, reproduction and brain development in many vertebrates including fishes, positively affecting their cognitive abilities. However, how n‐3 LC‐PUFA impact fish behaviour and cognition in natural habitats remains unclear. Populations and individuals of the same species often vary in their capacities to synthesise n‐3 LC‐PUFA due to their local adaptations and life‐history trade‐offs. This may affect their sensitivity to dietary intake of these nutrients and, in turn, their cognitive traits and ecological performance. Here, we tested how dietary n‐3 LC‐PUFA affects behavioural and cognitive traits of brown trout *Salmo trutta* from two lacustrine and three riverine populations. We combined laboratory behavioural tests with experiments in semi‐natural flume mesocosms to see how dietary treatment affects foraging behaviour in a natural environment (i.e., prey size and taxonomic composition in their stomach contents). Trout raised on a high n‐3 LC‐PUFA diet showed less bold behavioural types and better cognitive performance in laboratory tests and capacity to capture and consume larger prey in the flume mesocosm. Additionally, we observed interpopulation differences in behaviour and cognition, although these differences were independent of whether fish were from lakes or river.

## Graphical abstract

With this study, we tested the role of dietary n‐3 LC PUFA on behaviour and cognition of brown trout *Salmo trutta* from different lacustrine and riverine populations. Secondly, we tested the ecological relevance of these traits evaluating fish resource acquisition in a semi‐natural stream mesocosm through the inspection of stomach contents. We found that fish fed a high n‐3 LC PUFA diet had better cognitive performance, lower boldness and better capacities on catching larger prey. Behavioural and cognitive traits differed between population independently of whether trout were from lake or river.

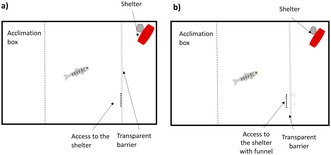

## 1 Introduction

Animal populations and individuals consistently differ in a suite of often‐correlated behavioural traits such as boldness (i.e., tendency of an individual to express risky behaviour), activity and aggressiveness (Réale et al. 2007). They can also differ in cognitive flexibility, which has been proposed to associate with these behavioural traits following the speed–accuracy trade‐off, where bold, active and aggressive individuals should be able to explore the environment faster but less efficiently and have lower cognitive flexibility (Sih and Del Giudice 2012). These behavioural traits are related to resource acquisition in the wild, which mediates the relationship between behaviour and fitness (i.e., survival and reproduction, Haave‐Audet et al. 2022). The theory predicts that consumers foraging on a diet rich in omega‐3 long chain polyunsaturated fatty acids (n‐3 LC‐PUFA) will have higher cognitive flexibility and show less bold (i.e., less prone to get engaged in risky situations), less active and less aggressive behavioural types and are able to explore the environment more efficiently (Závorka et al. 2023). Yet, these predictions are based on laboratory findings, and the importance of these nutrients for the development of ecologically important behavioural traits in the natural environment remains poorly understood.

Omega‐3 LC‐PUFA are a group of vital dietary biomolecules beneficial for the development of somatic and reproductive tissues (Bogevik et al. 2020; Yonar et al. 2020) and for neuronal functioning in vertebrates (Pilecky et al. 2021). For instance, in riparian insectivorous birds, n‐3 LC‐PUFA (i.e., DHA – docosahexaenoic acid and EPA – eicosapentaenoic acid) increase breeding success (Twining et al. 2018, 2019), while in fishes, the acquisition through maternal provisioning determines the antipredator performance of offspring (Fuiman and Ojanguren 2011; Perez and Fuiman 2015). The availability of these biomolecules across food webs depends on differences in the capacities of primary producers to synthetize them (Twining et al. 2021). Aquatic food webs have a high availability of n‐3 LC‐PUFA (Hixson et al. 2015; Twining et al. 2016), which also differs across aquatic food webs. Lacustrine systems are more enriched in these molecules than the riverine ones, which rely on both aquatic autochthonous resources and terrestrial allochthonous inputs (Brett et al. 2017). The uneven distribution of n‐3 LC‐PUFA across food webs can lead to adaptive intra‐specific differences among individuals and populations of the same species in capacity to internally synthesise n‐3 LC‐PUFA from molecular precursors (e.g., ALA – α‐Linolenic acid). For example, fish populations occupying different habitats show intraspecific endogenous differences in the synthesis of n‐3 LC‐PUFA (Ishikawa et al. 2021; Mock et al. 2019). Lacustrine three‐spined sticklebacks, which are zooplanktivorous, have a higher n‐3 LC‐PUFA dietary acquisition than river populations, which feed prevalently on macroinvertebrates (Hudson et al. 2022). Correspondingly, riverine sticklebacks have a higher copy number of the fads2 gene responsible for the desaturation of molecular precursors to counterbalance the dietary deficiency (Ishikawa et al. 2021). However, it is not clear how this variation affects the sensitivity of individuals to n‐3 LC‐PUFA intake and their behavioural and cognitive traits and eventually their ecological performance.

Brown trout ( *Salmo trutta* ) show a wide variation in behaviour (Johnsson and Näslund 2018), resource acquisition (Sánchez‐Hernández and Cobo 2018) and habitat use (Ferguson et al. 2019). Lake migratory populations of brown trout have a piscivorous diet, from which they acquire most of the n‐3 LC‐PUFA; on the other hand, the stream resident populations are insectivorous, and the amount of n‐3 LC‐PUFA is determined by both diet and internal synthesis (Murray et al. 2014; Syrjänen et al. 2011). These two ecotypes may differ in their sensitivity to dietary intake of n‐3 LC‐PUFA, which may generate differences in brain development (Závorka et al. 2022), and possibly also in behavioural and cognitive traits and ecological performance. However, the effects of dietary n‐3 LC‐PUFA on ecologically important behaviours across different ecotypes or populations of a plastic species such as brown trout have never been tested.

Here, we report the results of a common garden feeding experiment that investigated the role of dietary n‐3 LC‐PUFA in the development of brown trout behavioural traits and cognition and ecological performance. We tested activity, boldness, aggressiveness and performance in inhibitory control detour tests (ICDT) among individuals of brown trout originating from five populations and fed with high or low n‐3 LC‐PUFA diets. Additionally, we used flume mesocosms to assess the effect of dietary treatments on foraging behaviour in habitat and prey availability close to natural conditions. We expected that higher dietary n‐3 LC‐PUFA availability would result in better performance in ICDT and that enhanced cognitive skills of fish would result in a higher capacity to catch large‐sized prey items and detect cryptic benthic invertebrates in a semi‐natural flume mesocosm. Secondly, according to the speed‐accuracy trade‐off, we expected fish with enhanced dietary ICDT performance to be less bold, active and aggressive. Finally, we expected lacustrine brown trout to be more sensitive to an n‐3 LC‐PUFA deprived diet than stream residents, because stream residents should have a greater capacity to synthesise n‐3 LC‐PUFA.

## 2 Material and Methods

### 2.1 Fish Collection

In November 2022, we collected juveniles (age 0+) from four hatcheries maintaining genetic stocks of brown trout from different populations in Austrian freshwater systems (Drau River, Kamp River, Lake Attersee and Lake Weissensee) and from one wild population from the Ois River (Ybbs). All fish from hatcheries were 10 months old at the beginning of the experiment. The age of the wild fish collected in the river by electrofishing was estimated based on the length‐frequency distribution of the population of origin and corresponded to age 0+ and a fork length of 85.27 ± 8.52 mm. Each of the five populations was represented by *N* = 70 sampled individuals.

### 2.2 Laboratory Conditions

Fish were kept in flow‐through holding tanks (500 L) supplied with filtered spring water in a flow‐through system, with water temperature naturally fluctuating over time (depending on outdoor conditions) in a range suitable for growth and development of brown trout i.e., from 4°C in January to 15°C in July (Jonsson and Jonsson 2011) and maximal daily amplitude of ~0.5°C. Fish were fed daily *ad libitum* and the feed was supplied automatically between 9.00 and 16.00 by a feeding belt. The daily amount of pellet (GARANT AQUA, Austria) was distributed in 5 silicon cups placed and then released by the feeder.

### 2.3 Dietary Treatment and Holding Tanks Distribution

Fish were initially fed for 2 weeks by feed from their hatchery of origin and the wild fish by a mix of hatchery feeds. After this period, we distributed fish among 10 holding tanks, where each population was split between two tanks. One group from each population received rapeseed oil coated pellet feed with low n‐3 LC‐PUFA content, and the other received fish oil coated pellet feed with high n‐3 LC‐PUFA content. The experimental feeds were isonitrogenous and isocaloric and differed only in n‐3 LC‐PUFA composition; for detailed diet composition, see (Závorka et al. 2021). In February 2023, when all individuals apparently achieved the minimal size for tagging (i.e., 69 mm, Vollset et al. 2020), fish were anaesthetized (2‐phenoxyethanol, 0.5 mL·L^−1^), tagged with 12‐mm (PIT tags), and measurements of their fork length (distance from the tip of the snout to the end of the central caudal fin ray) to the nearest millimetre and body mass to the nearest 0.1 g were taken (Table 1). This allowed individual identification of fish throughout the experiment. After tagging, individuals were randomly re‐distributed among the holding tanks to minimise the effect of the holding tank differences on the experimental results and to reduce the collinearity between the population of origin and the holding tank. A second redistribution among the holding tanks was done before fish were transferred from the holding tanks to the flume mesocosm. At each of these redistributions, we maintained the dietary treatment of individuals, so fish fed high n‐3 LC‐PUFA diet were always redistributed only among the tanks with this dietary treatment, and the same logic applied for the low n‐3 LC‐PUFA dietary treatment. In both of these cases, we kept a maximum of 35 individual fish per 500 L holding tank. We selected a total of 256 individuals for behavioural scoring and kept the remaining fish as replacements in case of the death of some experimental individuals. Mortality of fish during captivity was low and consistent with the previous long‐term feeding studies conducted in the husbandry facility (Murray et al. 2014). Dead individuals from the experimental holding tanks were replaced by individuals from the additional tanks with similar body size and the same population of origin and dietary treatment. For more details of the distribution of individuals among the holding tanks, see the diagram in Figure S1.

**TABLE 1:** Summary table of fish body weight and fork length for all the populations.

Population of origin	Ecotype of origin	Fork length after HyTEC Flume (mm)	Body weight after HyTEC Flume (g)	*n* spring	*n* summer	*n* HyTEC flume
Kamp	River	105.16 ± 12.98	15.62 ± 18.43	54	37	20
Drau	River	137.23 ± 18.82	28.71 ± 11.02	52	47	22
Ois	River	113.12 ± 13.83	16.87 ± 6.81	49	33	21
Attersee	Lake	138.40 ± 12.74	31.14 ± 8.78	52	50	21
Weissensee	Lake	127.29 ± 23.48	25.61 ± 17.48	54	32	17

*Note:* Lengths and body weights shown in the table are the ones taken after the HyTEC Flume experiment. Together with these, there are the number of fish tested in laboratory behavioural and cognitive tests for each population in spring and summer. Some fish were tested both in spring and summer; thus, the two groups overlap. Finally, the number of fish tested in the HyTEC Flume is reported.

### 2.4 Behavioural and Cognitive Scoring in Laboratory Conditions

To evaluate the variation and the temporal consistency of behaviour and cognition of individuals, we tested each fish in six sets of scoring trials (*T* = 6) focused on their behavioural types (i.e., boldness, activity and aggressiveness) and cognitive skills. Three sets of trials were conducted in spring (March–April 2023) and three in summer (May–mid August 2023). Fish were scored in batches using 16 rectangular plastic tanks (*W* = 56 cm, *L* = 77 cm, *H* = 30 cm, water depth ~15 cm), cleaned and filled with fresh freshwater after each trial to account for potential interference by olfactory cues. All the features in the scoring tanks necessary for the individual test were remotely controlled by a set of strings allowing synchronised actions in all 16 tanks (e.g., hoisting of the guillotine door of the acclimation box) without fish disturbance (Figure S2). Scoring tanks were positioned underneath cameras recording the trials. Behavioural metrics were extracted from the video records by automatic video tracking analysis. We tracked fish behaviour using a YOLOv3 model with the OpenCV package in Python (script available at https://github.com/P‐Hansson/trout‐tracking‐and‐behaviour). Videos showing issues during recording, and trials with failed or corrupted video records were excluded from the analysis. In total, we have extracted behaviour from 3094 individual behavioural trials.

Behavioural trials for scoring of boldness in Emergence Test (Budaev 1997), activity in Open Field Test (Réale et al. 2007), aggressiveness in Mirror Image Test (Näslund and Johnsson 2016), and cognitive skills in transparent barrier detour test (Kabadayi et al. 2018) were performed in a sequence without removal of the fish from the scoring tank (similar to (Závorka et al. 2020)), but there were slight differences in the sequence of the behavioural trials between spring and summer.

**FIGURE 1:**
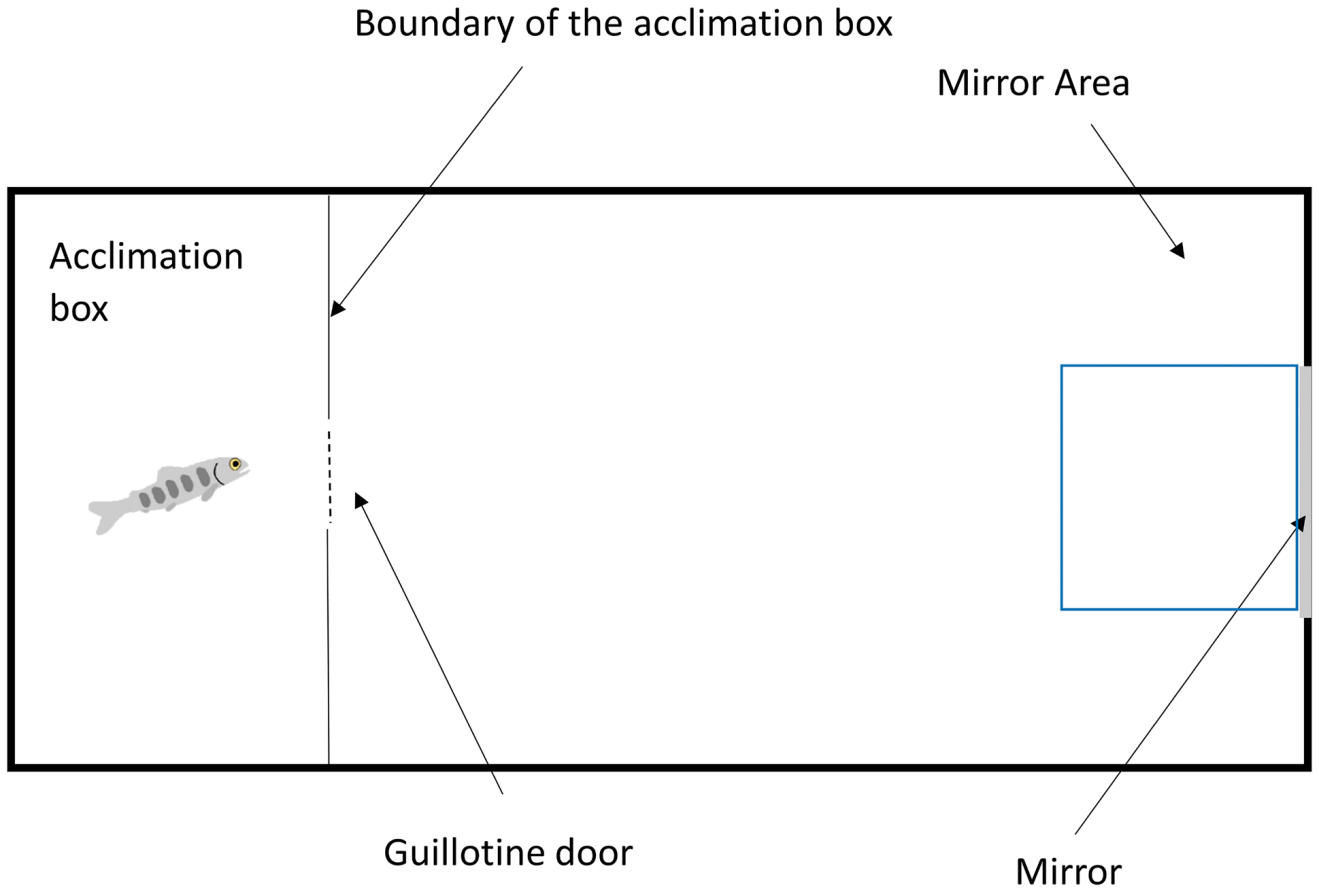
Representation of all apparatus in the behavioural scoring in laboratory condition Fish were tested in plastic scoring boxes (*W* = 56 cm, *L* = 77 cm, *H* = 30 cm). Before the test they waited into an (*W* = 56 cm, *L* = 20 cm, *H* = 30 cm) acclimation box for 15 min. The dashed line highlights the opening of the guillotine door to allow the emergence from the acclimation box. After the Emergence Test trial ended (duration of the trial: 15 min) we lifted up the acclimation box and started with the Open Field Test for 20 min. Fish was test to see the space it could cover through swimming expressed in cm. After the Open Field Test we inserted a guillotine mirror (20 × 20 cm^2^) and Mirror Image Test started. Area selected valuable for interaction between experimental subject and mirror image (blue‐sky outline square) was 19.75 × 20 cm^2^.

During the behavioural scoring in the spring, individuals were initially scored in sets of trials focused on their behavioural type. The sequence started with the Emergence Test when the fish was placed in the acclimation box of a scoring tank for 15 min (*W* = 56 cm, *H* = 30 cm, *L* = 20 cm, Figure 1). We then hoisted the guillotine door of the acclimation box and measured the time until emergence from the box to assess boldness (i.e., time until emergence in sec) of individuals. Individuals were given 15 min to emerge. Hereafter, regardless of whether an individual had emerged from the acclimation box, we hoisted the acclimation box from the scoring tank and left the individual in a barren tank to assess their activity (i.e., distance moved in cm) in the Open Field Test for 20 min. Afterwards, we inserted a mirror (20 × 20 cm) along the short wall of the scoring tank and recorded for another 10 min the aggressiveness of individuals towards their mirror image (i.e., time in sec that the individual spent in the zone 20 × 20 cm in front of the mirror) (Näslund and Johnsson 2016). After this trial, the fish were removed from the scoring tank, their PIT‐tag ID was recorded, and they were placed back in their holding tank. Each individual was scored three times in this sequence of trials with 1 week between the sequences.

**FIGURE 2:**
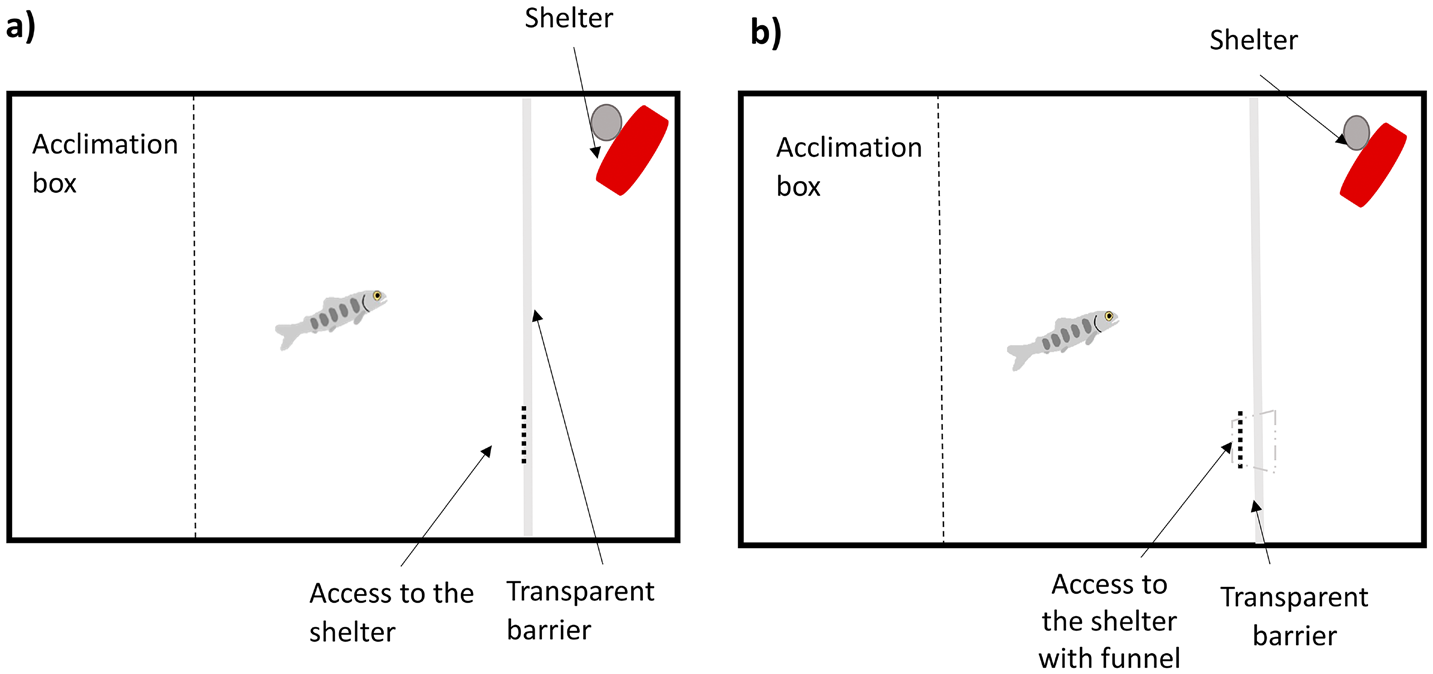
Representation of Inhibitory Control Detour Test (ICDT) apparatus performed in laboratory conditions. (a) Experimental set up of the simple version of the ICDT. Transparent barrier separated the experimental subject from the pipe/shelter. Fish could swim through one circular hole with 5 cm of diameter as the only possible access to reach the reward (dash‐line). (b) Experimental set up of the complex version of the ICDT. Transparent barrier separated the experimental subject from the pipe/shelter Fish could swim through one circular hole with 5 cm of diameter as the only possible access to reach the reward (dash‐line). An additional funnel in the access was insert (grey dash‐lines) to avoid that fish could pass the barrier through thigmotaxis.

After 3 weeks of scoring of behavioural traits, individuals were tested for their cognitive skills in the Inhibitory Control Detour Test (ICDT) trials. This cognitive test challenges the inhibition of predominant motor response towards a reward (Kabadayi et al. 2018). ICDT trials took place in the same scoring boxes as the trials for the assessment of behavioural types of the fish. The setup consisted of an acclimation box and a transparent acrylic barrier which divided the quarter of the scoring tank opposite to the acclimation box from the rest of the scoring tank (Figure 2). Behind the transparent barrier was an artificial shelter (an opaque PVC pipe [22 cm length, 4.5 cm diameter] attached to a stone), which served as a reward, motivating the fish to pass the barrier. The transparent barrier blocked the shortest direct access from the acclimation box to the reward. The reward was accessible only through an opening in the transparent barrier positioned on the side away from the reward. In the first ICDT trial in the spring, there was the opening in the transparent barrier without other features, but in the second and third trials, we fitted the opening in the transparent barrier with a transparent 8 cm long funnel (Figure 2b). The funnel increased the difficulty of the test for the fish, because they could not reach the opening in the barrier by simply swimming along the transparent barrier (i.e., thigmotaxis, ‘wall‐hugging’), but instead, the fish needed to move away from the barrier and find the opening of the funnel. During the ICDT trial, fish were placed for 10 min in the acclimation box to recover from handling. After this period, the whole acclimation box was hoisted and individuals were given 30 min to pass the transparent barrier and reach the reward (time in sec to pass the barrier was recorded). After the trial, fish were moved back to their holding tank. Each individual was scored three times in the ICDT trial with 1 week between the trials.

In summer, we tested fish again in both behavioural traits trials and ICDT trials. The difference compared to the sequences in the spring was that ICDT trials were conducted immediately after the behavioural traits trials without removing the fish from the scoring tanks. After the last behavioural traits trial, we placed the acclimation boxes back in the scoring tank and gently guided the fish to the box. We then changed the setup of the scoring tank for the ICDT trial, leaving individuals for 10 min in the acclimation box before the start of the ICDT trial. In summer, we fitted the transparent funnel to the opening of the barrier only in the third ICDT trial, while the first two trials were without the funnel. Each individual was scored three times in summer, but they were given only 3 days to recover between the trials. After the last summer trial, individuals were transported to the HyTEC Flumes.

### 2.5 Semi‐Natural Flumes Mesocosm Experiment

To evaluate the role of dietary treatment on ecological performance (i.e., resource acquisition) of the fish, we used semi‐natural flume mesocosms at the so‐called HyTEC facility in Lunz (HyTEC Hydro morphological and Temperature Experimental Channels, HyTEC) (Auer et al. 2017). The HyTEC Flumes simulate the conditions of a subalpine stream including natural flow conditions, substrate and prey. It consisted of two artificial channels each split into four equivalent enclosures (*N* = 8, *L* = 7 m, *W* = 1.5 m). Each enclosure was separated by a 2 m buffer zone enriched with stones and wood in order to decrease the speed of the flow and delay the invertebrates washing out. Enclosure dividers had a wooden barrier that could be used to regulate the water level in each enclosure. An acclimation pond for fish was placed at the downstream end of the two channels. Prior to the experiment, flumes were inoculated with macroinvertebrates collected from the Seebach River (47°51′23.00″ N, 15°02′12.44″ E; 47°51′08.14″ N, 15°03′52.46″ E): we collected 48 Multi‐Habitat samples (MHS), each made up of 20 subsamples of 25 × 25 cm^2^ sediment. From the MHS macroinvertebrate samples, 6 MHS samples were supplied to each Flume enclosure. We estimated an initial density of ~7500 macroinvertebrate individuals per square meter in the source stream, resulting in an estimated density of 5000–6000 prey items per square meter (or an estimate total of 55,000 prey items per enclosure) which corresponded to densities found in nature (Brown and Brussock 1991; Leitner et al. 2015) (Table S1). We periodically checked that the density of the prey in the HyTEC Flume remained at natural levels and repeated the inoculations when density decreased.

Before the experiment, fish were transferred from the husbandry laboratory facility to the flumes and placed for 7 days in the acclimation pond. In total, six focal brown trout of the same size and population of origin were released into each of the eight enclosures (*N* = 48). Individuals were distributed among the enclosures according to their feeding treatment and population of origin, so each enclosure contained individuals from the same population, where three individuals received an n‐3 LC‐PUFA‐rich diet (*N* = 3) and three received an n‐3 LC‐PUFA‐poor diet (*N* = 3), respectively. The experiment in the flumes lasted for 10 consecutive days. During this time, we manipulated the water level in the flume so there was a high level (day 1–4), low level (day 4–7) and high level (day 7–10), to simulate the water level fluctuation in a natural stream. One fish per enclosure was removed after the seventh day of the rearing to be used in another study (Austad et al. unpublished). On the 10th day (end of the experiment), fish were overdosed with 2‐phenoxyethanol (1 mL·L^−1^) measured for fork length and body mass, and their stomach was flushed by injecting water into their oesophagus through a syringe (Kamler and Pope 2010). Fish were then euthanized by cutting their spinal cord. Stomach contents were stored in 15 mL vials containing 90% ethanol in a freezer at −20°C until further processing.

### 2.6 Stomach Content Analysis

To measure dry biomass of the stomach content, we first oven‐dried 47‐mm diameter filter papers at 50°C for at least 24 h and measured individually their weight to the nearest mg. Then we offloaded the vial with stomach content and ethanol onto the filter paper using a vacuum filtration and kept the samples inside Petri dishes. Samples were then freeze‐dried (Genesis Freeze Dryer, Virtis, NYC) for 48 h and weighed. Dry biomass of the stomach content was calculated by subtracting the mass of the empty filter paper from the mass of the filter paper with stomach content. After this, we counted the number of prey items on each filter and determined them to the lowest possible taxonomic level (Table S1). For our analysis, we then calculated the average size of the prey item by dividing the total dry biomass by the number of prey items in the stomach, and we calculated the ratio between the number of prey items corresponding to terrestrial prey and aquatic benthic prey.

### 2.7 Statistical Analysis

We analyse the effects of n‐3 LC‐PUFA dietary treatments on behavioural traits and cognitive measures from ICDT in laboratory and stomach content measures collected in the HyTEC Flumes. All statistical analyses were done in R version 4.4.1 (R Core Team, Vienna, Austria).

#### 2.7.1 ICDT

The effect of the n‐3 LC‐PUFA dietary treatment on the time before fish solved the ICDT was initially tested with a mixed linear model (LMM) using the dietary treatment (categorical variable with two levels), the population of origin of fish (categorical variable with five levels), the numerical order of the test (categorical variable with three levels: 1st, 2nd and 3rd trial), and the season when they were tested (spring or summer, see Supporting Information) as fixed factors and Fish ID as a random effect. Season was included in the model because the spring protocol tested the complex ICDT design twice, while the summer protocol tested it only once. Only fish that passed the transparent barrier were used in this model. Another model was used to test the effects on the success rate in the ICDT (binary measure of whether fish passed or not the transparent barrier), but since it did not converge even after scaling and centring model variables, it could not be interpreted. We included only measurements from the version of the ICDT with no funnel in our data analysis, as the version of the ICDT had a very low success rate and produced a zero‐inflated dataset (Figure S4).

#### 2.7.2 Behavioural Types: Boldness, Activity and Aggressiveness

We tested the effect of the n‐3 LC‐PUFA dietary treatment on fish boldness, activity and aggressiveness using their respective measures in LMM as dependent variables with the dietary treatment, population, interaction dietary treatment: population and test order as fixed factors and Fish ID as a random factor. In all of these models, the interaction between dietary treatment and population was not significant. We thus ran LMMs again without including any interaction between fixed factors (Table 4). For the evaluation of boldness, we also ran a generalised linear mixed model (GLMM) for binomial logit distribution using the same structure of fixed and random factors as for LMM, but with a binary dependent variable (1 – emerged or 0 – not emerged from the shelter). Since all the behavioural tests were run in the same way both in spring and summer, we did not run a single model with season as a cofactor but rather designed one model for each season.

Repeatability of an individual's performance in both behavioural traits' trials and ICDT was tested as the variance of the random intercept of an individual's ID adjusted to the fixed factors used in the main models described above (Stoffel et al. 2017). We used 1000 bootstraps to calculate the 95% confidence interval (CI) for repeatability estimates to see the consistency of performances across trials (Nakagawa and Schielzeth 2010). We considered a trait moderately repeatable when its repeatability was between 0.3 and 0.4 and its lower confidence interval was higher than zero, and thus it is significant (Bell et al. 2009).

#### 2.7.3 Stomach Content Measures

For the measures of stomach content from the HyTEC Flume experiment, we used GLMM for binomial logit distribution to see the effect of dietary treatment on the proportion of benthic prey versus drifting prey and LMM for the effect of dietary treatment on average prey size and prey biomass. For both of the two responses, the fixed factors were the interaction of dietary treatment and population, the sex of the fish and scaled values of their fork length. We included the fork length of the fish in these models, but not for the behavioural and cognitive measures from the laboratory, because the final size of individuals was measured only after the HyTEC Flume experiment, and thus it did not reflect the size of the fish during the laboratory scoring several months earlier. Similarly, we included sex as a fixed factor only in the model from HyTEC flumes, because this information was available only for the subset of individuals that were tested in the mesocosms and not for all the fish tested for behaviour in the laboratory. We used as a random effect the combination of the round of the HyTEC flume experiment fish took part in (from 1st to 10th round) and the enclosure of the HyTEC flume where fish were placed (from 1st to 8th enclosure of the flume) to account for the possible spatial–temporal variability of the environment in the flume enclosures (Table 5).

The significance of the response variables of the fitted models was evaluated through an ANOVA (Type II sum of squares or type III for models without and with interactions, respectively) using the car package from R. Differences among trials on the ICDT and personality tests were analysed using Tukey's HSD post hoc test. Response variable latencies to solve the ICDT and prey size were log transformed for the fit of the models that were evaluated by visually inspecting the normality distribution of the model's residuals. All models reported in the results have satisfactorily met the evaluated criteria for their robustness.

## 3 Results

### 3.1 N‐3 LC‐PUFA Dietary Treatment and ICDT

**FIGURE 3:**
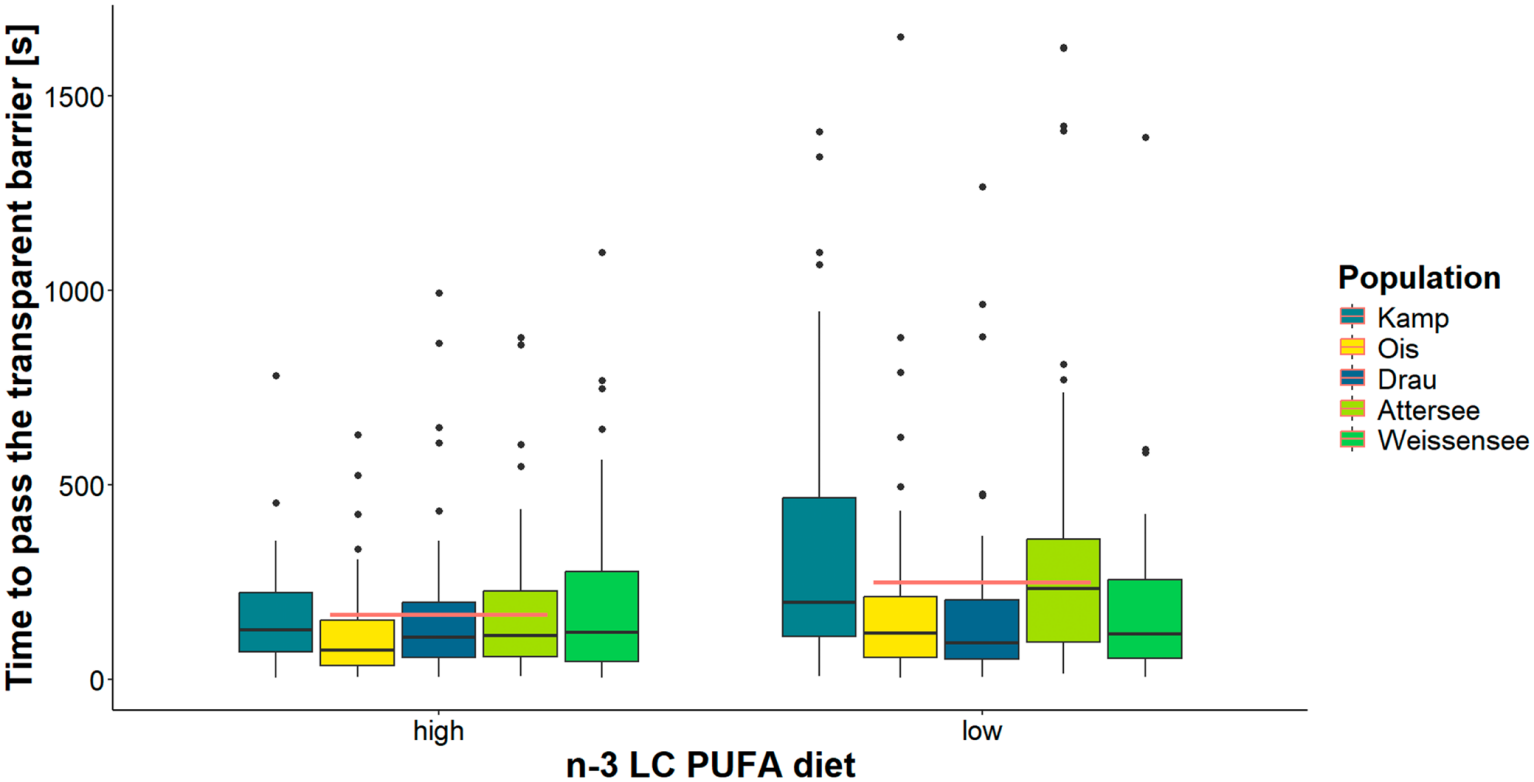
Effect of n‐3 LC‐PUFA dietary treatment on inhibitory control detour test. The box plot displays the time (in seconds, logarithmic scale) required to pass the transparent barrier for both the high and the low n‐3 LC PUFA dietary treatment. The five colours group for fish's population of origin: Kamp (teal blue), Ois (neon yellow), Drau (deep blue grey), Attersee (bright lime) and Weissensee (light green). The black dots outside of the boxplots are the outlier measures. Each treatment group has five box plots representing each a population, showing the median, quartiles and potential outliers.

The measure of fish ICDT performance (time of latency before the experimental subject passed the barrier) was affected by the n‐3 LC‐PUFA dietary treatment (Table 2, *F* = 8.95, *p* = 0.003). Fish that received a high n‐3 LC‐PUFA dietary treatment took less time to pass the barrier (88.7 ± 8.56 s) than the low n‐3 LC‐PUFA treatment group (121.4 ± 11.00 s) and were therefore approximately 27% faster in solving the ICDT (Figure 3). An effect was also found for the population of origin, with fish from the Ois River having performed better than fish from the Drau River and Lake Weissensee (*F* = 4.29, *p* = 0.002, Figure 3). Post hoc Ois River vs. Drau River and Lake Weissensee *p* < 0.05; the rest of the *p* = NS. Additionally, season (spring or summer) was associated with ICDT performance, with fish performing better in spring than in summer (*F* = 3.94, *p* = 0.047). Repeatability for the performance in ICDT was very low at 0.0899 (95% CI: 1.47e^−16^−0.189).

**TABLE 2:** Landscape table of all the estimates from behavioural and cognitive tests: For each taken measure, there are mean ± SE. Estimates are grouped by dietary n‐3 LC PUFA content (high or low), population (Kamp, Ois, Drau, Attersee and Weissensee) and season (spring or summer) when is needed.

Season	Behavioural measures	High n‐3 LC PUFA	Low n‐3 LC PUFA	Kamp	Ois	Drau	Attersee	Weissensee	Spring	Summer
Spring and Summer	Latency time (s)–ICDT	88.7 ± 8.56	121.4 ± 11.00	137.0 ± 18.3	76.4 ± 10.3	92.7 ± 11.7	129.1 ± 15.9	96.0 ± 12.9	73.3 ± 16.3	139.1 ± 14.8
Spring	Time to leave the shelter (s)–Emergence test (boldness)	175 ± 13.4	206 ± 14.5	196 ± 22.2	211 ± 27.3	178 ± 19.2	181 ± 21.4	187 ± 20.4		
	Emerged/not emerged–Emergence test (boldness)	0.802 ± 0.036	0.832 ± 0.034	0.765 ± 0.058	0.607 ± 0.077	0.917 ± 0.029	0.789 ± 0.055	0.895 ± 0.036		
	Distance (cm)–Open field test (activity)	5699 ± 131	5568 ± 132	5106 ± 204	4405 ± 216	6639 ± 206	6043 ± 208	5976 ± 204		
	Time in front of the mirror (s)–Mirror image test (aggressiveness)	165 ± 7.26	173 ± 7.20	163 ± 11.2	126 ± 12.1	167 ± 11.3	253 ± 11.3	135 ± 11.3		
Summer	Time to leave the shelter (s)–Emergence test (boldness)	210 ± 15.5	163 ± 14.6	248 ± 29.2	151 ± 23.4	217 ± 22.9	182 ± 20.7	142 ± 22.6		
	Emerged/not emerged–Emergence test (boldness)	0.955 ± 0.033	0.965 ± 0.029	0.953 ± 0.042	0.941 ± 0.053	0.981 ± 0.018	0.935 ± 0.052	0.971 ± 0.030		
	Distance (cm)–Open field test (activity)	7317 ± 215	7162 ± 225	6345 ± 359	6820 ± 378	8265 ± 317	6790 ± 306	7978 ± 389		
	Time in front of the mirror (s)–Mirror image test (aggressiveness)	137 ± 7.88	130 ± 8.23	148.6 ± 13.2	110.8 ± 13.8	131.0 ± 11.6	198.5 ± 11.2	77.9 ± 14.2		

### 3.2 N‐3 LC PUFA Dietary Treatment and Boldness

The Emergence Test (time to emerge from the shelter) was not affected by any of the considered variables during spring but showed differences for all of them in summer (Table 3). In summer, fish that received a high n‐3 LC‐PUFA dietary intake had higher latency times to emerge than the latency times of those fed on a low n‐3 LC‐PUFA diet instead. Latency times were not different between the first and the second trial, but they were between the first and the third (Post hoc first Emergence Test trial vs. third *p* < 0.05). Fish from Lake Weissensee took less time than those from Kamp River in leaving the shelter (Post hoc Lake Weissensee vs. Kamp River *p* < 0.05). Repeatability of the Emergence Test was low, corresponding to 0.130 (95% CI: 0.014–0.233) during spring and 0.280 (95% CI: 0.164–0.384) during summer.

**TABLE 3:** Summary model of Inhibitory Control Detour Test (ICDT).

Response	Fixed effect	F	Df	Df.res	Pr (>*F*)	Random effects		
Latency time before passing the transparent barrier (log transformed)	Population	4.29	4	221.47	0.0023*	(1|fish_ID)		
	Dietary n‐3 LC‐PUFA	8.95	1	221.56	0.003*	**R**	**SE**	**CI**
						0.089	0.049	1.47e^−16^‐0.189
	Test order	0.495	2	470.72	0.61			
	Season	3.945	1	563.99	0.047*			

*Note:* *p < 0.05; **p < 0.01; ***p < 0.001.

The success rate in the Emergence Test was significantly affected by the population of origin (*χ* ^*2*^ = 20.34, *p* < 0.001) in spring, with fish from River Ois performing the Emergence Test worse than fish from Lake Weissensee and Drau River (post hoc Ois vs. Drau and Weissensee *p* < 0.05 and the rest *p* = NS, Table 4). In summer, there was a tendency for the numerical order of the trial (*χ* ^*2*^ = 5.822, *p* = 0.054) on the probability of leaving the shelter rate in the Emergence Test. Fish tended to emerge less on the third last trial compared to the prior ones (post hoc 1st trial vs. 2nd and 3rd trials *p* < 0.05). No effects on the probability of leaving the shelter in the Emergence Test were found from dietary treatment and population (Table 4). Individual repeatability of the probability of leaving the shelter in the Emergence Test was moderate, corresponding to 0.323 (95% CI: 0.064–0.154) in spring and 0.617 (95% CI: 0.149–0.452) in summer.

**TABLE 4:** Summary models of behavioural scoring from spring and summer experiments.

Season	Behavioural type	Response	Fixed effect	*χ* ^2^	Df	*P* (> *χ* ^2^)	Random effect		
Spring	Boldness	Latency to emerge from the shelter (square root transformation)	Dietary n‐3 LC‐PUFA	2.46	1	0.117	(1|fish_ID)		
			Population	1.25	4	0.869	**R**	**SE**	**CI**
							0.13	0.55	0.014–0.233
			Test order	0.42	2	0.808			
		Emerged/not emerged	Dietary n‐3 LC‐PUFA	0.46	1	0.497	(1|fish_ID)		
			Population	20.34	4	< 0.001***	**R**	**SE**	**CI**
							0.323	0.064	0.154–0.406
			Test order	0.474	2	0.789			
	Activity	Distance (cm)	Dietary n‐3 LC‐PUFA	0.497	1	0.487	(1|fish_ID)		
			Population	69.77	4	< 0.001*	**R**	**SE**	**CI**
							0.606	0.033	0.537–0.665
			Test order	21.52	2	< 0.001***			
	Aggressiveness	Time in front of the mirror (s)	Dietary n‐3 LC‐PUFA	0.66	1	0.418	(1|fish_ID)		
			Population	77.72	4	< 0.001***	**R**	**SE**	**CI**
							0.289	0.042	0.201–0.369
			Test order	2.54	2	0.281			
Summer	Boldness	Latency to emerge from the shelter (square root transformation)	Dietary n‐3 LC‐PUFA	4.964	1	0.026*	(1|fish_ID)		
			Population	12.66	4	0.013*	**R**	**SE**	**CI**
							0.280	0.057	0.164–0.384
			Test order	7.252	2	0.027*			
		Emerged/not emerge	Dietary n‐3 LC‐PUFA	0.197	1	0.657	(1|fish_ID)		
			Population	2.84	4	0.585	**R**	**SE**	**CI**
							0.617	0.149	0.452–0.936
			Test order	5.82	2	0.054			
	Activity	Distance (cm)	Dietary n‐3 LC‐PUFA	0.249	1	0.617	(1|fish_ID)		
			Population	23.69	4	< 0.001***	**R**	**SE**	**CI**
							0.615	0.035	0.542–0.678
			Test order	3.07	2	0.216			
	Aggressiveness	Time in front of the mirror (s)	Dietary n‐3 LC‐PUFA	0.35	1	0.554	(1|fish_ID)		
			Population	52.09	4	< 0.001***	**R**	**SE**	**CI**
							0.5	0.043	0.416–0.579
			Test order	3.86	2	0.145			

*Note:* *p < 0.05; **p < 0.01; ***p < 0.001.

### 3.3 N‐3 LC PUFA Dietary Treatment and Activity

**FIGURE 4:**
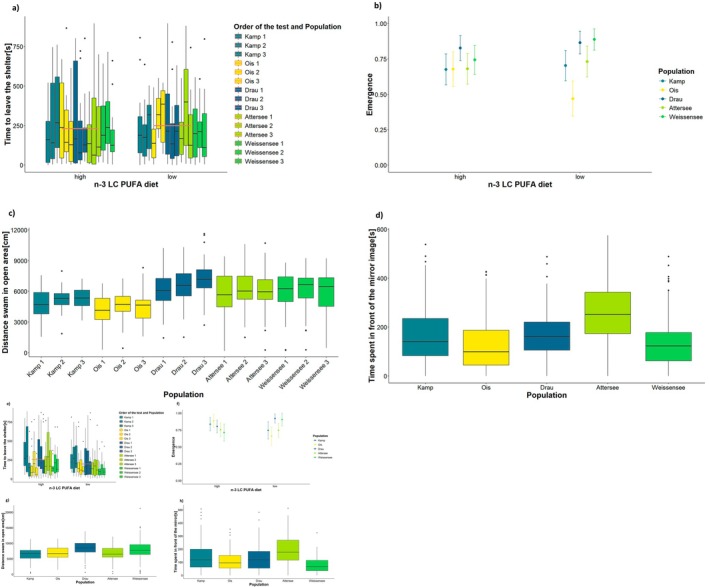
Influences of variables on behavioural measures in spring (a–d) and summer (e–h). (a–e) Effect of n‐3 LC‐PUFA on boldness (latency time). The boxplots compare the time passed before fish left the shelter (*y*‐axis) in the five populations (colour legend) under the two dietary treatments (*x*‐axis). The black dots outside of the boxplots are the outlier measures. Each dietary treatment group has 15 box plots (5 population × 3 behavioural trial) representing the high and low n‐3 LC‐PUFA treatments, showing the median, quartiles and potential outliers. The coral line across the box plots represents the averaged measures from the two dietary groups. (b–f) Effect of n‐3 LC‐PUFA on boldness (binary measure on whether fish emerged or not from the shelter). The scatter plot with error bars compares the emergence success (*y*‐axis) for fish populations (Kamp, Ois, Drau, Attersee, Weissensee) under high and low n‐3 LC‐PUFA dietary treatments (*x*‐axis). Each dietary treatment group has 5 corresponding points grouping each population of origin, showing variability within the groups. (c–g) Differences between populations in fish Open Field Test performance (activity). The boxplot shows the distance in centimetres swam in open arena (*y*‐axis) by different fish populations (*x*‐axis). The black dots outside of the boxplots are the outlier measures. Each population has one box plots representing the population of origin, showing the median, quartiles and potential outliers. Specifically for the spring plot (c), there are also indicated the repeated trials along the experiment. Each population, thus have three boxplots for each trial. (d–h) Differences between populations in fish Mirror Image Test performance (aggressiveness). The boxplots compare the time spent in front of the mirror (in seconds) by fish from various populations (Kamp, Ois, Drau, Attersee, Weissensee). Each population has one boxplot showing the median, quartiles and potential outliers.

Activity of fish in Open Field Test was significantly affected by population (*χ* ^2^ = 69.77, *p* < 0.001) and test order (*χ* ^2^ = 21.52, *p* < 0.001) in spring, while there were no differences between the two dietary treatment groups. Fish were less active in the 1st trial compared to the 2nd and the 3rd ones (post hoc 1st trial vs. 2nd and 3rd trials *p* < 0.05). Riverine fish from Drau showed a similar level of activity as lacustrine fish from Attersee and Weissensee, while fish from Kamp River and Ois River were less active than the rest of the populations (Figure 4b). The effect of population on fish activity was significant also in the summer model (*χ* ^*2*^ = 23.69, *p* < 0.001, Figure 4e), whilst there was no effect of dietary treatment (*χ* ^*2*^ = 0.249, *p* = 0.617) nor of test order (*χ* ^*2*^ = 3.07, *p* = 0.216). Fish from Drau River remained the most active compared to all the other populations (post hoc Drau vs. all the rest *p* < 0.05), while fish from lake Weissensee were more active than those from Kamp River (post hoc Weissensee vs. Kamp *p* < 0.05); activity among the rest of the populations did not differ. Repeatability for activity was high, being 0.606 (95% CI: 0.537–0.665) in spring and 0.615 (95% CI: 0.542–0.678) in summer.

### 3.4 N‐3 LC PUFA Dietary Treatment and Aggressiveness

Dietary treatment did not affect the time fish spent in front of the mirror (spring: *χ* ^2^ = 0.66, *p* = 0.418; summer: *χ* ^2^ = 0.35, *p* = 0.554). Time spent by fish in front of the mirror was affected by the population both in spring (*χ* ^2^ = 77.72, *p* < 0.001) and in summer (*χ* ^2^ = 52.09, *p* < 0.001). Particularly, fish from Lake Attersee spent more time in front of their mirror image compared to the other populations (post hoc Attersee vs. other population *p* < 0.01) (Figure 4c and Figure 4f). Individual repeatability of aggressiveness was moderate, being 0.289 (95% CI: 0.201–0.369) in spring and 0.500 (95% CI: 0.416–0.579) in summer.

### 3.5 N‐3 LC PUFA Dietary Treatment and Resource Acquisition

**FIGURE 5:**
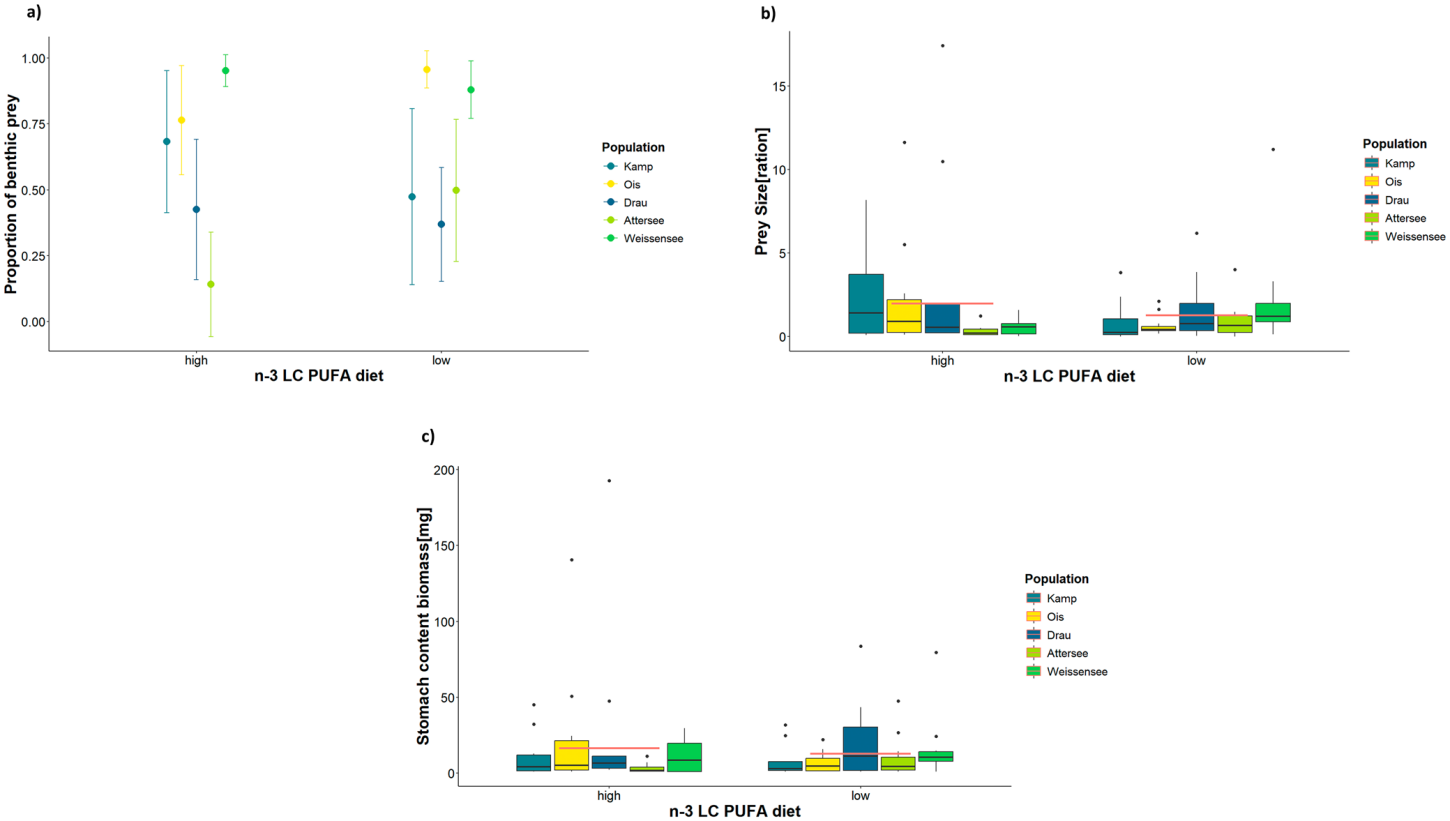
Effect of n‐3 LC‐PUFA dietary treatment on dietary intake in HyTEC Flumes. (a) Effect of dietary n‐3 LC‐PUFA on acquisition of benthic prey versus drifting prey. The scatter plot with error bars shows the proportion of benthic versus drifting prey (*y*‐axis) in the diet of fish from five populations (Kamp, Ois, Drau, Attersee, Weissensee) under high and low n‐3 LC‐PUFA dietary treatments (*x*‐axis). Each population has two points and a vertical line, indicating confidence intervals. (b) The boxplot compares prey size (logarithmic scale) on y‐axis for five fish populations (Kamp, Ois, Drau, Attersee, Weissensee) under high and low n‐3 LC‐PUFA dietary treatments (*x*‐axis). Each population has two box plots representing the high and low n‐3 LC‐PUFA treatments, showing the median, quartiles and potential outliers (black dots outside the boxplot). The coral line across the box plots represents the averaged measures from the two dietary groups. (c) Effect of n‐3 LC‐PUFA dietary treatment on the acquisition of biomass. The box plot compares the dry stomach content biomass on the *y*‐axis (logarithmic scale) for fish populations (Kamp, Ois, Drau, Attersee, Weissensee) under high and low n‐3 LC‐PUFA dietary treatments (*x*‐axis). Each population has two box plots representing the high and low n‐3 LC‐PUFA treatments, showing the median, quartiles and potential outliers (black dots outside the boxplot). The coral line across the box plots represents the averaged measures from the two dietary groups.

We found an effect of the dietary n‐3 LC‐PUFA treatment on the prey size that fish have been selecting in the flume mesocosms (*χ* ^*2*^ = 5.35, *p* = 0.021). Fish that received a high dietary n‐3 LC‐PUFA treatment fed on larger prey than the low dietary n‐3 LC‐PUFA treatment conspecifics (Figure 5b). We also found a tendency for the interaction between population of origin and dietary n‐3 LC‐PUFA treatment (*χ* ^*2*^ = 9.46, *p* = 0.051). This tendency reveals some variation between fish population capability on prey size when fed with different dietary treatments. Fish coming from lacustrine populations (Attersee and Weissensee) and fish from the Drau River conditioned to a low n‐3 LC‐PUFA diet fed on larger prey compared to the ones under the high n‐3 LC‐PUFA dietary treatment (Figure 5b). We found no effects of any variable considered on total prey biomass (Table 5). Fish across populations differed in the proportion of benthic and drifting prey they consumed (*χ* ^*2*^ = 10.06, *p* = 0.039). Fish from Ois fed more on benthic prey than fish from Lake Attersee and the Drau River (post hoc Ois vs. Attersee and Drau *p* < 0.05, Figure 5a). There were no effects of dietary treatment, sex and their fork length on the proportion of benthic prey.

**TABLE 5:** Summary models of stomach content measures.

Response	Fixed effect	Random effect	*χ* ^2^	Df	Pr(> *χ* ^2^)
Proportion benthic prey versus drifting prey	Dietary n‐3 LC‐PUFA	(1|enclosure:round)	2.17	1	0.141
	Population of origin		10.06	4	0.039*
	Sex		0.05	1	0.831
	Scaled fish fork length		1.42	1	0.233
Prey size (log transformed)	Dietary n‐3 LC‐PUFA	(1|enclosure:round)	5.35	1	0.021*
	Population of origin		5.66	4	0.226
	Sex		0.01	1	0.939
	Scaled fish fork length		2.43	1	0.112
	Population: Dietary treatment		9.46	4	0.051
Stomach content dry biomass (log transformed)	Dietary n‐3 LC‐PUFA	(1|enclosure:round)	0.228	1	0.633
	Population of origin		3.24	4	0.519
	Sex		0.454	1	0.500
	Scaled fish fork length		1.438	1	0.230

*Note:* *p < 0.05; **p < 0.01; ***p < 0.001.

## 4 Discussion

Our results showed that n‐3 LC‐PUFA dietary intake increased fish cognitive performance in the ICDT. Differences in this performance between the five populations suggest that they may differ in their cognitive abilities and responses to the laboratory environment. However, fish cognitive performance and their response to dietary treatment were not affected by whether fish came from a lake or a river. The dietary treatment had no effect on aggressiveness and activity, but it affected one of the two boldness measures (i.e., latency time), with the high n‐3 LC‐PUFA dietary group taking more time to leave the shelter. Additionally, we found that fish fed on a high n‐3 LC‐PUFA diet caught larger prey compared to individuals fed the low n‐3 LC‐PUFA diet when exposed to natural prey in HyTEC Flume mesocosms. Finally, we found that the n‐3 LC‐PUFA dietary treatment had no effect on the acquisition of more benthic prey compared to the drifting ones.

Inhibitory control is an ecologically relevant cognitive process improving foraging skills in birds (Coomes et al. 2022) and guaranteeing survival in mammals (Rochais et al. 2023). Fishes' ICDT performances are comparable to the ones found in birds and mammals (Lucon‐Xiccato et al. 2017). However, the ecological relevance for this group had never been empirically tested (Lucon‐Xiccato 2024; Lucon‐Xiccato et al. 2017). Brown trout inhibitory control may favour a better evaluation of the environment and selection of higher quality prey. In our study, fish with better inhibitory control performances showed higher foraging efficiency (i.e., high n‐3 LC‐PUFA dietary treatment group fed on larger prey). This suggests that laboratory ICDT is ecologically relevant for the evaluation of fish cognition. Whilst in general the high n‐3 LC‐PUFA diet group fed on larger prey, the effect of n‐3 LC‐PUFA on prey size tends to differ between populations. Fish from the lacustrine population (Lake Weissensee and Lake Attersee) fed on smaller prey when they were fed with a high n‐3 LC‐PUFA diet, while fish populations from streams (Kamp, Drau and Ois Rivers) fed on larger prey in the same dietary treatment conditions (i.e., high n‐3 LC‐PUFA diet). In the Atlantic bluefin tuna larvae ( *Thunnus thynnus* ), DHA, the most important n‐3 LC‐PUFA, promoted the synthesis of opsin and improved catching on rotifers prey (Koven et al. 2018). In the retina, DHA in cell membranes' phospholipids changes the conformation of photopigments so that they can bind with G‐Proteins, causing an improvement in the elaboration of visual stimuli (Mitchell et al. 2003). According to these mechanisms, we propose that an enrichment in dietary n‐3 LC‐PUFA (i.e., DHA) may have positively affected the visual acuity of trout from lake ecotypes, improving their sight and making them capable of better detecting and catching smaller prey. Our analysis revealed that the n‐3 LC‐PUFA dietary treatment did not influence the proportion of benthic versus drifting prey in trout diets. However, several factors must be considered when interpreting these results. The HyTEC Flume facility, being an open system, was subject to environmental variables that could influence prey community composition and availability. Contribution of benthic prey in trout diet increased with time (Figure S5). Possibly, the availability of the drifting prey, which included prey with an aerial imago stage, was more available in the early weeks of the experiment and decreased over time when the main period of insect emergence was over. Repeatability for ICDT performance was very low. We suggest that this value may indicate a high level of cognitive flexibility rather than low consistency or reliability of the measures (Nakagawa and Schielzeth 2010) since, with repeated trials for the same test, some fish may have learned how to solve the task in a reduced amount of time, increasing the variation in cognitive performance measures (Cauchoix et al. 2018).

The latency time to leave the shelter in the Emergence Test (boldness) performed in spring did not differ between the dietary treatment groups in any variables, but the measures collected in summer differed between all the variables, dietary treatment, population and test order. Fishes' behavioural types can emerge later across their lifetime during the passage from juvenile to subadult and after their sexual maturation (Polverino et al. 2016). This is because within‐individual behavioural variation (i.e., behavioural plasticity) is age‐dependent and tends to decrease the more time passes, increasing in turn the consistency of behavioural types (Fischer et al. 2014). In trout we tested, differences in boldness for dietary treatment, population and test order may have come out later in their development due to the age dependence of behavioural plasticity. In another salmonid species – the arctic charr (*Salvelinus alpinus*) – the level of boldness relied more on between population variation than on whether fish are surface or benthic feeders (i.e., feeding modalities) in determining behavioural types (Dellinger et al. 2023). However, the authors of this study provided the same feed to both the benthic and surface feeding groups, not considering the effect of diet biochemical composition of the different prey that benthic and surface feeders find in nature. Fish from the high n‐3 LC‐PUFA dietary group were less bold than the low n‐3 LC‐PUFA group. Since they are also the ones that performed better in ICDT, these results confirm our hypothesis on the speed‐accuracy trade‐off, with less bold individuals having higher cognitive performance than the low n‐3 LC‐PUFA bolder group.

Variation in behavioural types in brown trout is often heritable (Kortet et al. 2014). We suggest that the population genetic difference of brown trout in our study could play a role in determining the behavioural traits of boldness (time to leave the shelter and success rate from our experiment), activity and aggressiveness. Activity and aggressiveness did not differ between the dietary treatment groups. Prior studies in different animal taxa (i.e., invertebrates) showed that several behavioural types are affected by a surplus or a decrease of carbohydrates and/or proteins in their diet (Han and Dingemanse 2015). n‐3 LC‐PUFA are components of phospholipids (polar lipids) involved in determining biological structures such as biological membranes and their features (Quinn et al. 1989). On the other hand, carbohydrates function in energy storage. The same function is also performed by neutral lipids (i.e., triacylglycerol). Additionally, in southern field cricket, it was found that a high protein diet made individuals more aggressive (Han and Dingemanse 2017). Thus, we suggest that macronutrients involved in energy provisioning (i.e., carbohydrates and/or neutral lipids) are the determinants for fish activity and proteins for aggressiveness. Our experiment could not test this effect as our dietary treatment consisted of giving fish an isocaloric diet, which differed only in n‐3 LC‐PUFA content. The repeatability of behavioural measures we took from our experiments ranged from moderate to high both in spring and summer. These values confirmed the consistency of these behavioural types across time and contexts.

Fish activity in the Open Field Test tended to increase on the second trial done in spring and stayed at similar levels on the third one. Trout may be less active in the first trial of the experiment because of the lack of habituation to the new experimental conditions. This habituation effect on fish may also explain differences in the Open Field Test between spring and summer, with 3 out of 5 populations (River Mur and lakes Attersee and Weissensee) shown to be the most active in spring, and only one of these three resulting in being consistently active in summer (River Mur) (Stamps and Groothuis 2010). Finally, the habituation could also be the reason for the loss of significant differences between spring and summer emergence success rate measures in the Emergence Test and the decrease of latency time in the third trial of the same test.

In summary, our study demonstrates that dietary n‐3 LC‐PUFA could play an important role in the development of fish cognitive skills and foraging efficiency (i.e., catching larger prey) in natural habitat. Our findings showed the positive influence of n‐3 LC‐PUFA on cognitive skills and the negative effect on fish boldness in non‐model species and confirmed the ecological relevance of laboratory ICDT for the evaluation of cognition and the speed‐accuracy trade‐offs. Future studies could dive deeper and highlight the inner physiological mechanisms improved by n‐3 LC‐PUFA dietary intake on cognitive performance and the interpopulation differences in sensitivities for these molecules to highlight both proximal and distal causes of the development and evolution of fish cognition.

## Author Contributions

**Stefano Mari:** conceptualization (equal), data curation (equal), formal analysis (equal), methodology (equal), writing – original draft (lead), writing – review and editing (lead). **Stefan Auer:** investigation (equal), methodology (equal), resources (equal), writing – review and editing (equal). **Benedikte Austad:** investigation (equal), methodology (equal), resources (equal), writing – review and editing (equal). **Pernilla Hansson:** data curation (equal), investigation (equal), methodology (equal), software (lead), writing – review and editing (equal). **Simon Vitecek:** investigation (equal), methodology (equal), writing – review and editing (equal). **Mourine J. Yegon:** investigation (equal), methodology (equal), writing – review and editing (equal). **Libor Závorka:** conceptualization (equal), funding acquisition (lead), investigation (equal), methodology (equal), project administration (lead), resources (equal), supervision (lead), writing – review and editing (equal).

## Conflicts of Interest

The authors declare no conflicts of interest.

## Supporting information


**Data S1:** ece372340‐sup‐0001‐supinfo.docx.

## Data Availability

The data for this study are archived and publicly accessible on the FigShare platform at the following link: https://doi.org/10.6084/m9.figshare.28428659.v1.
