# Supplementary material for: Dietary Omega‐3 Long‐Chain Polyunsaturated Fatty Acids Can Enhance Ecologically Relevant Cognitive Traits in Juvenile Brown Trout
Source: Ecol Evol. 2025 Oct 16;15(10):e72340. doi: 10.1002/ece3.72340 (PMC12530006; doi:10.1002/ece3.72340)
Supplement: Supplementary file 1 — Data S1: ece372340‐sup‐0001‐supinfo.docx. [file ECE3-15-e72340-s001.docx]

***Supplementary material***


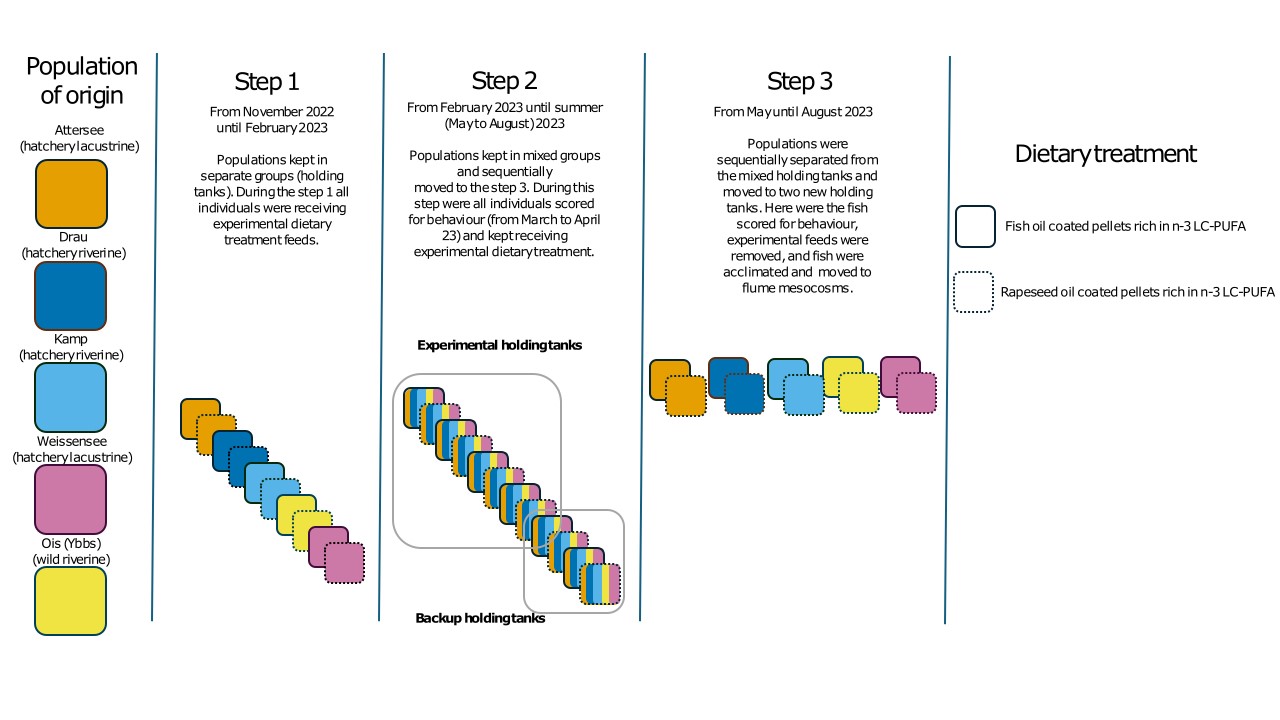


***Figure S1:*** *a) Diagram representation of the distribution of experimental fish in the holding tanks; fish coming for different population are represented with a square differently coloured. Outline of each square differ according to the dietary treatment (full line; high n-3 LC-PUFA, dash line; low n-3 LC PUFA).*

**c)**

**b)**

**a)**

***
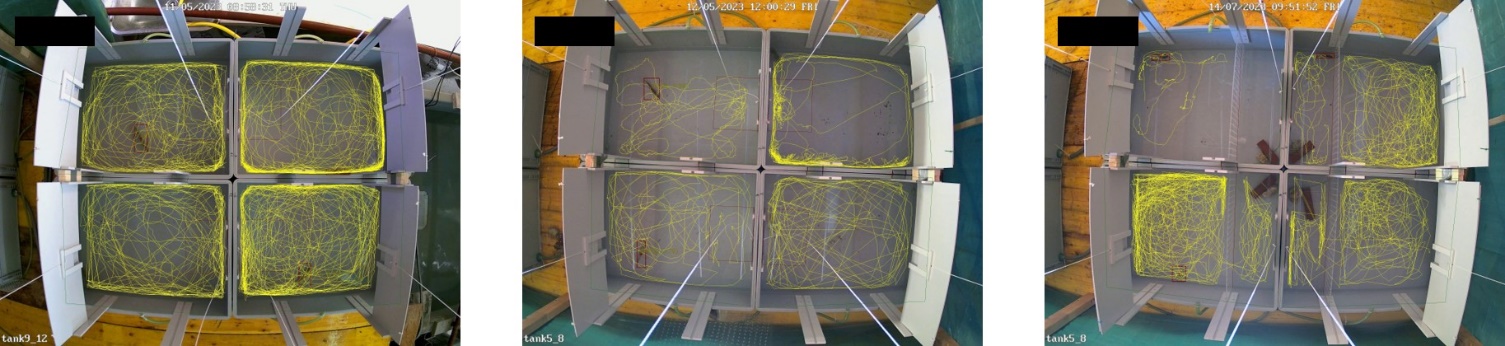
***

***Figure S2:*** *Screenshots of video tracking of open field test (a), mirror image test (b) and inhibitory control detour test (ICDT, c).*

Buffer zone enriched with wood and stones

Barrier for separation and water height change

Enclosure

**Water flow direction**


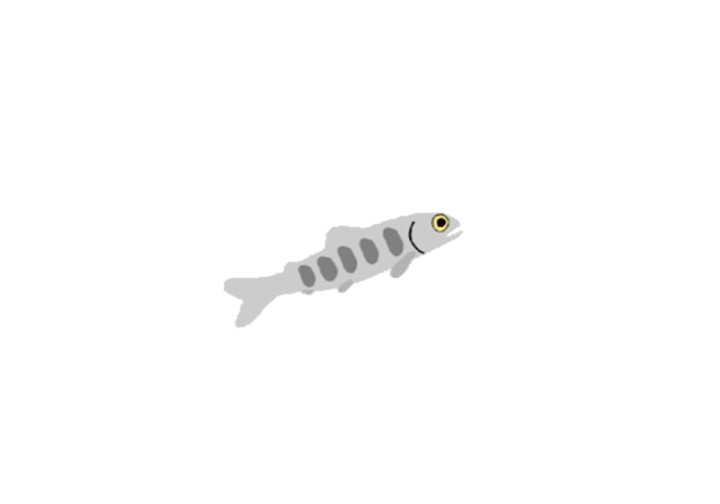

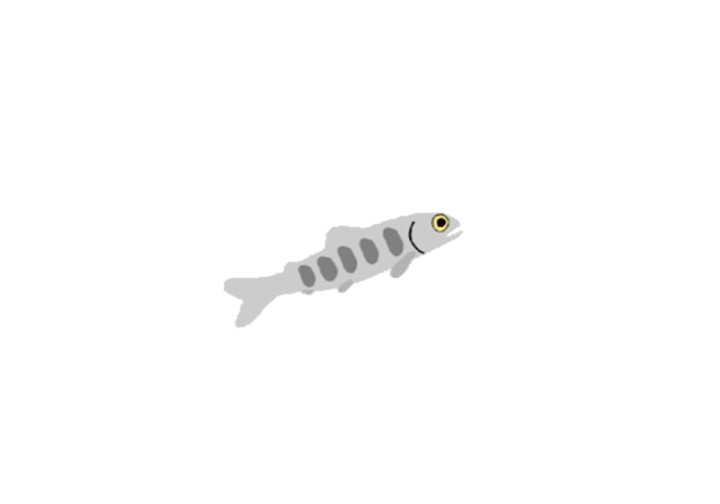

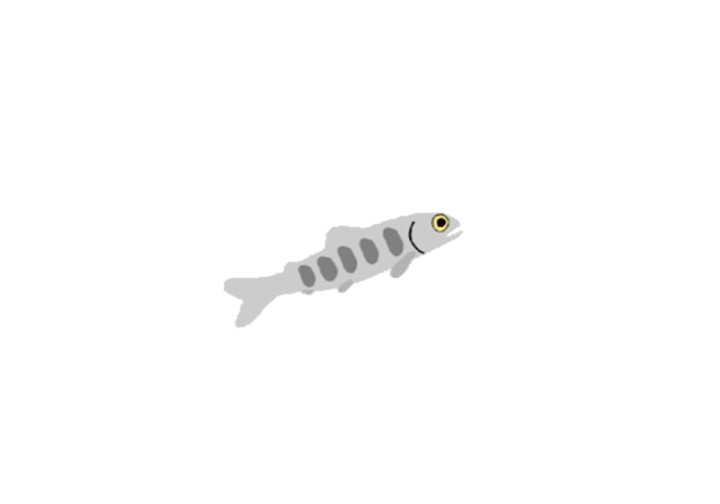

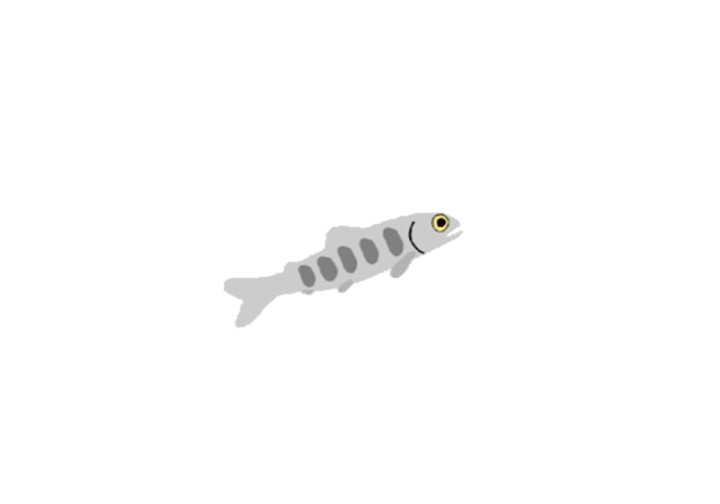

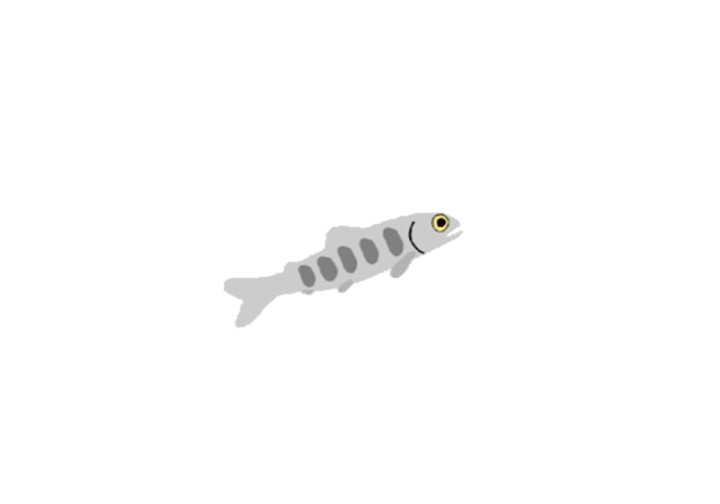

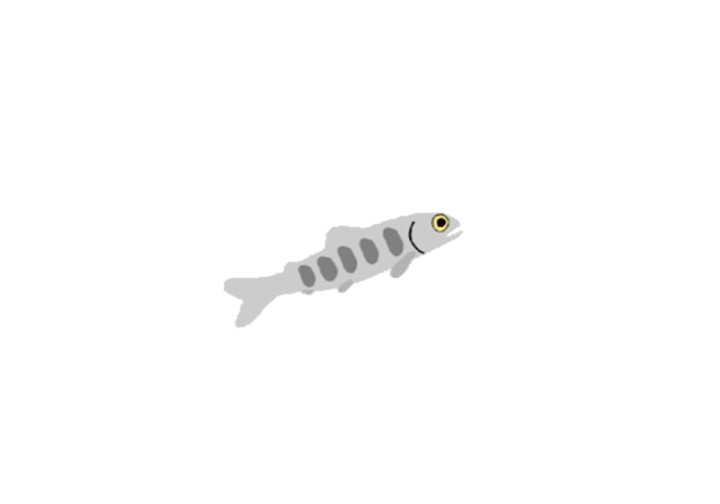

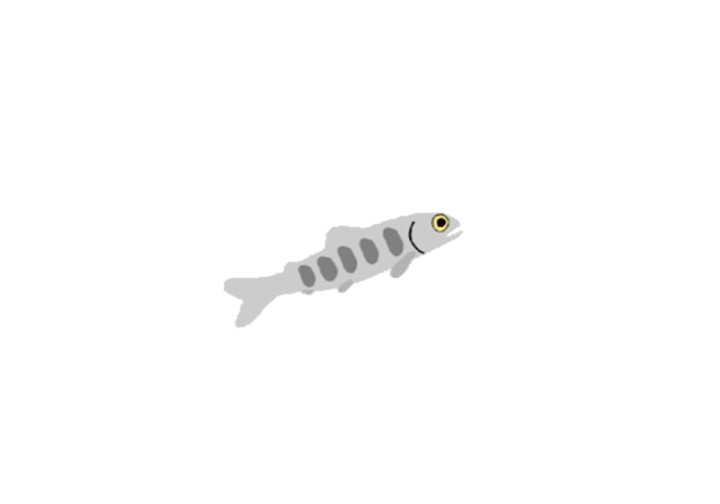

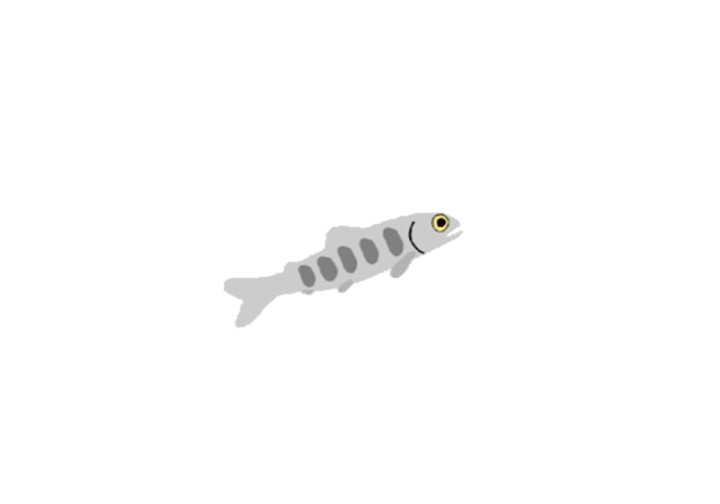

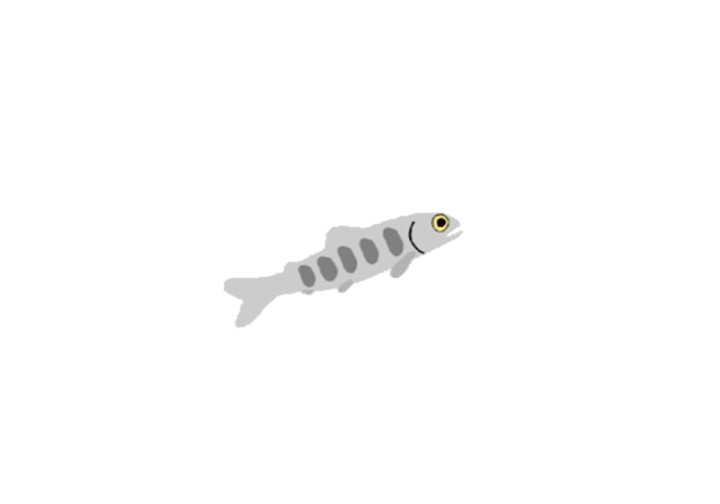

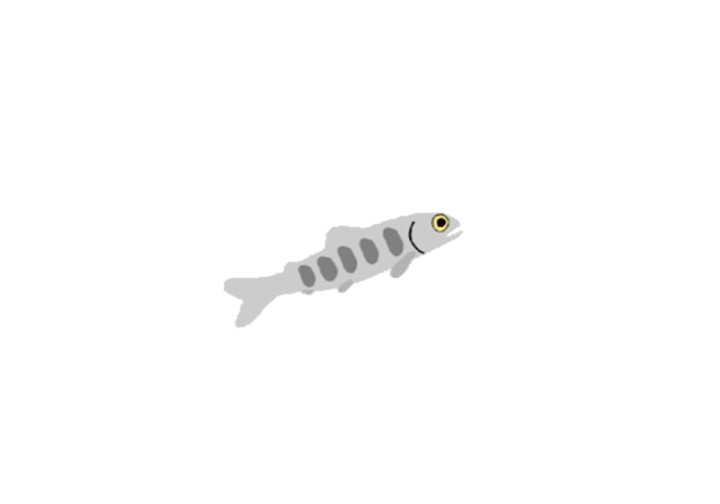

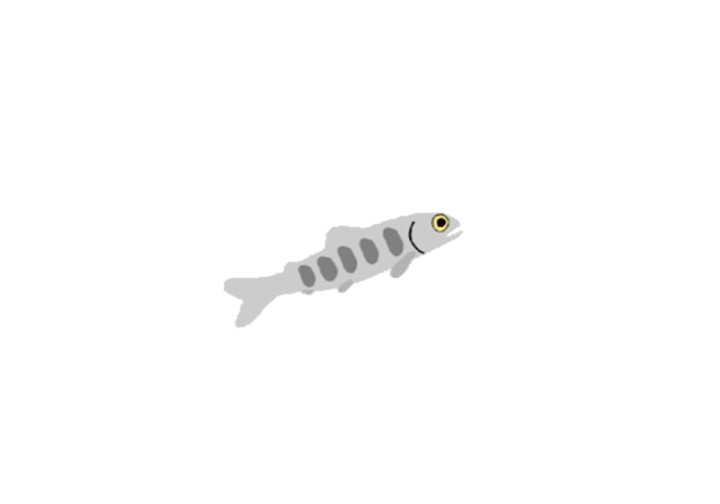

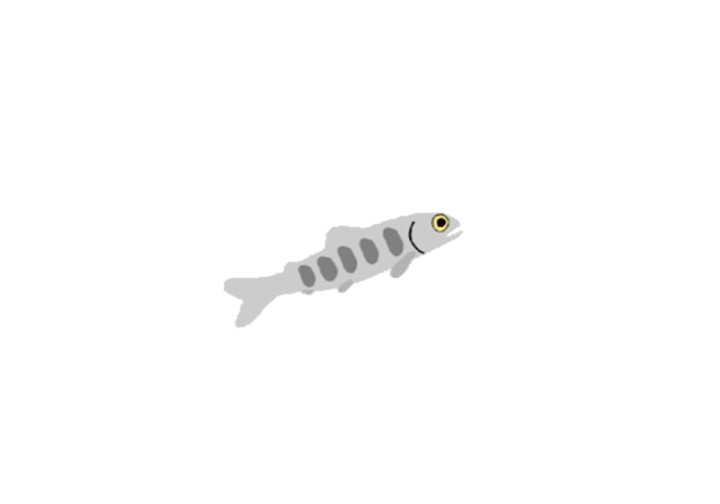

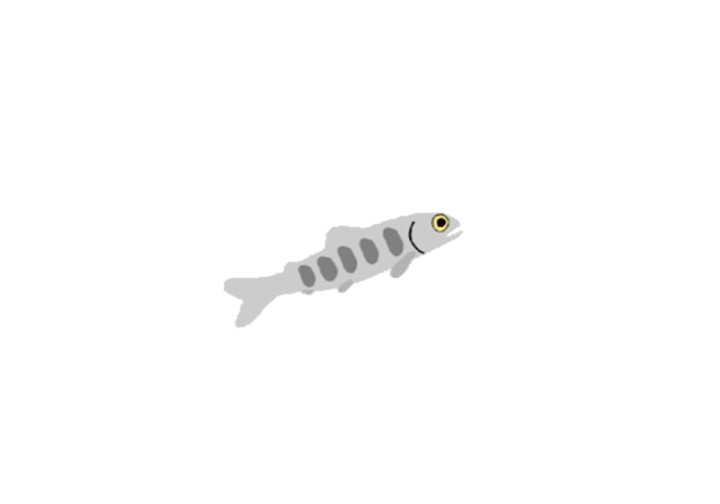

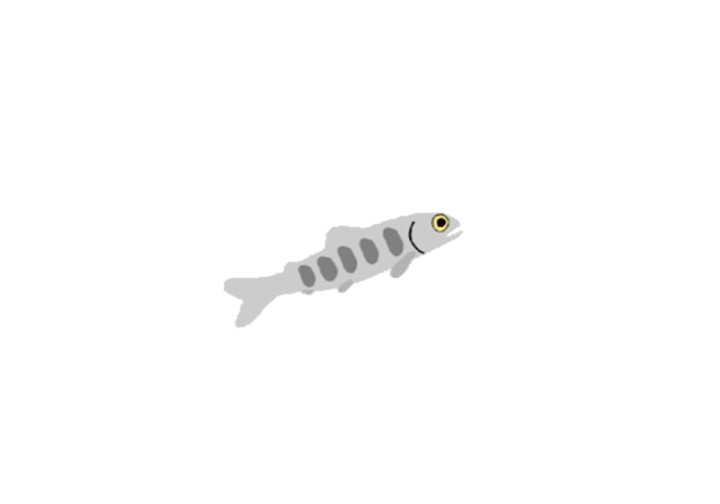

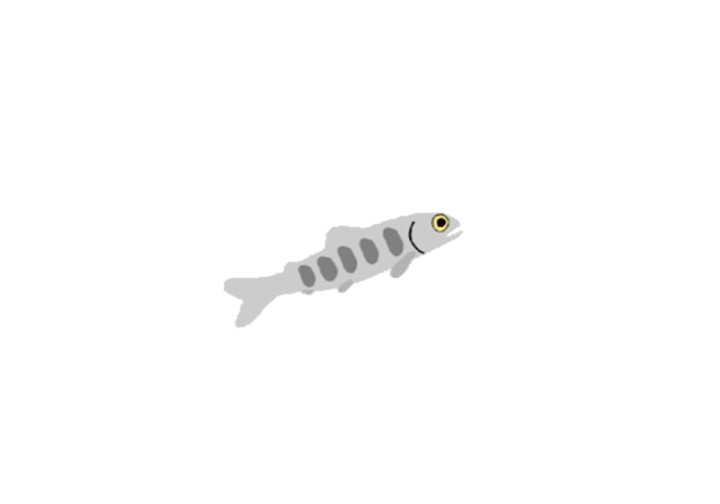

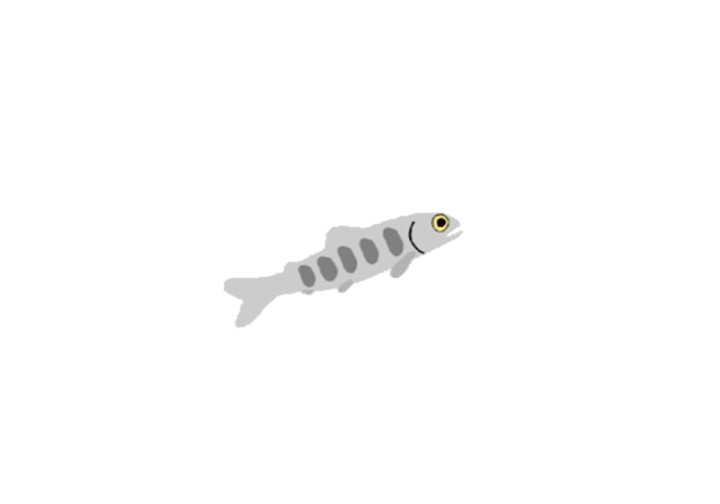

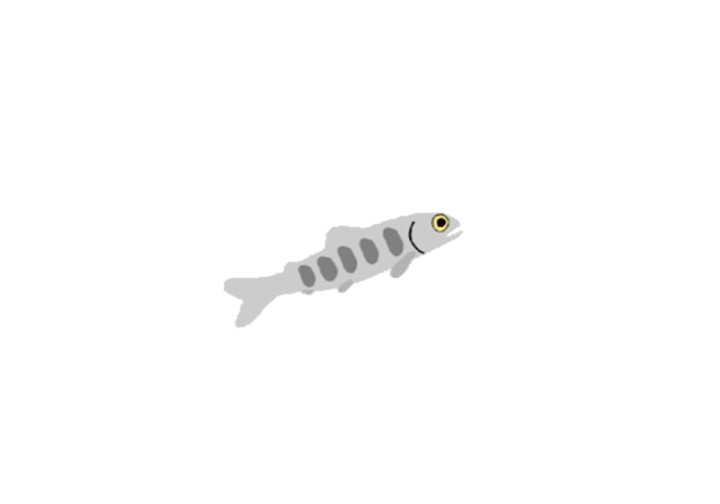

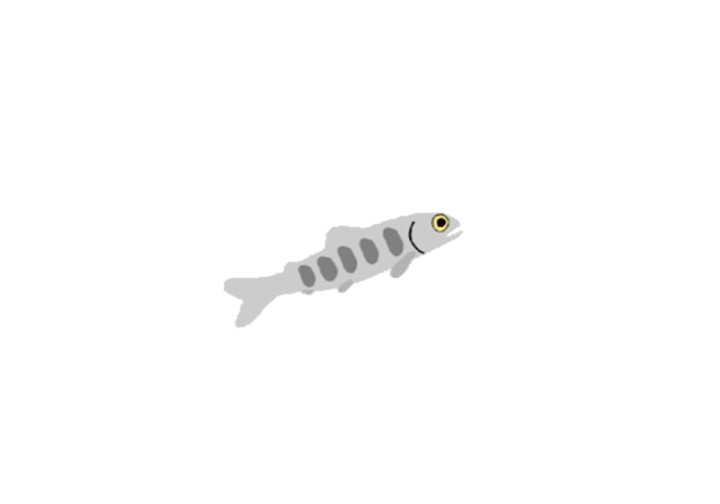

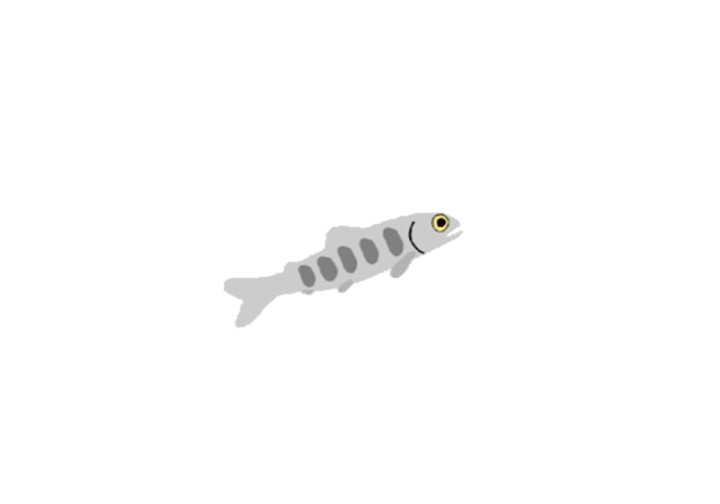

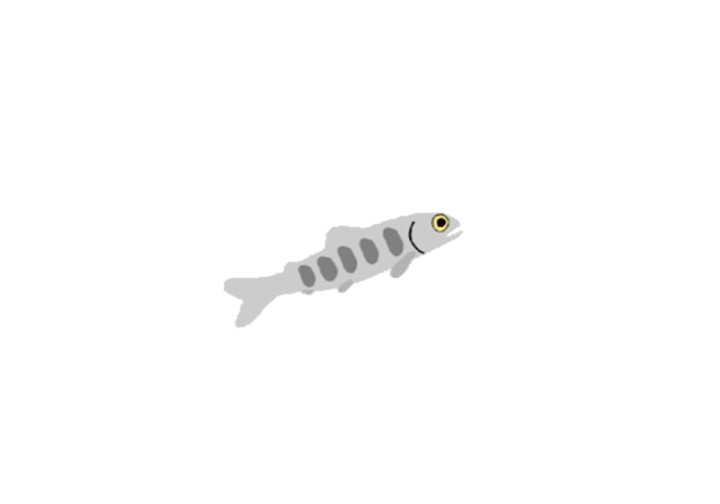

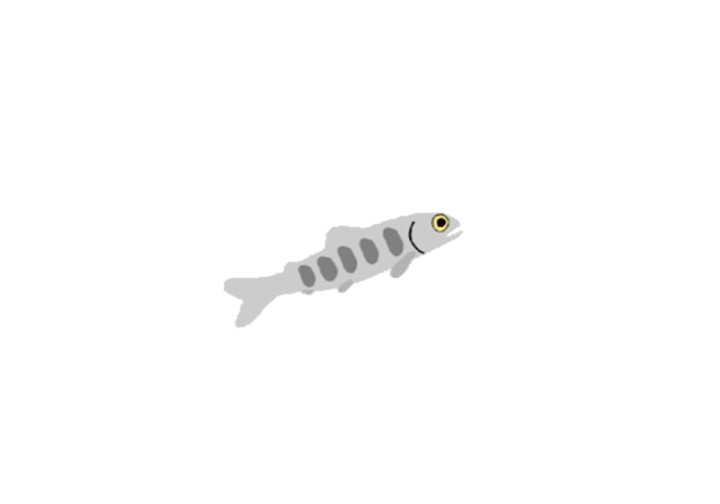

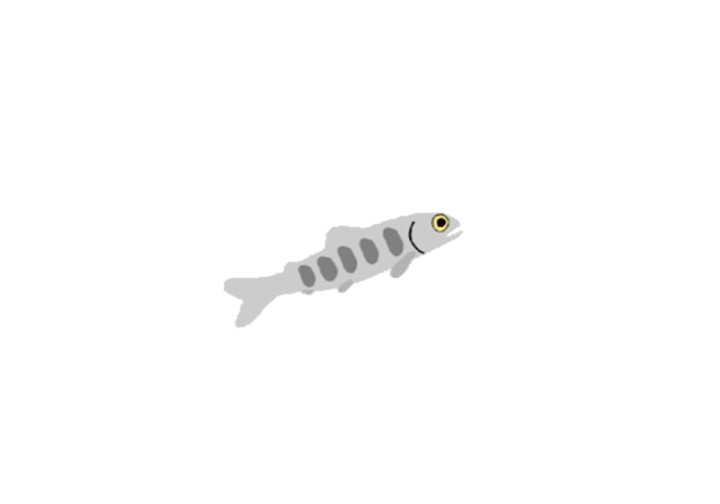

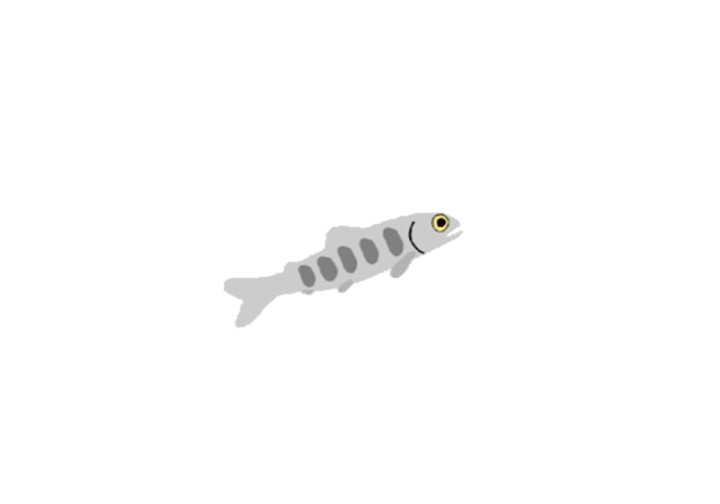

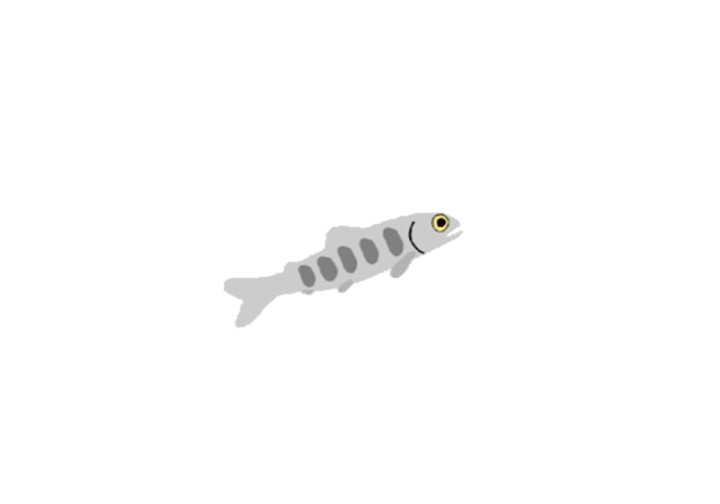

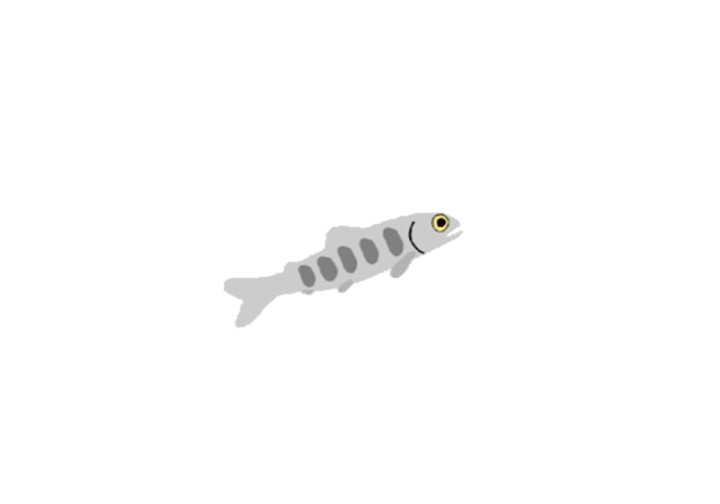

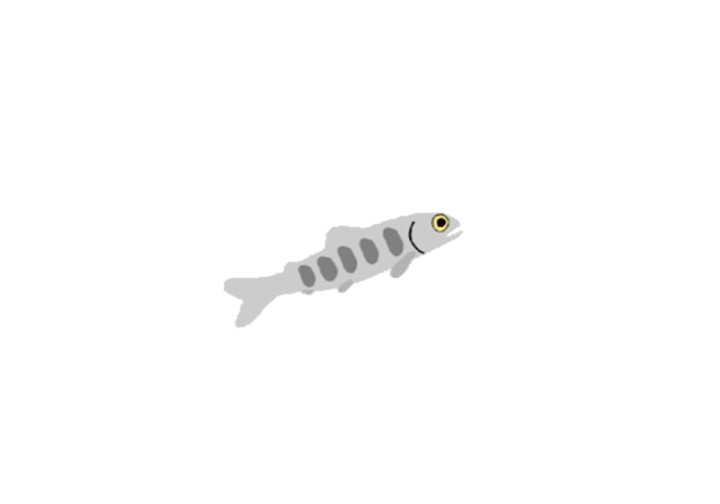

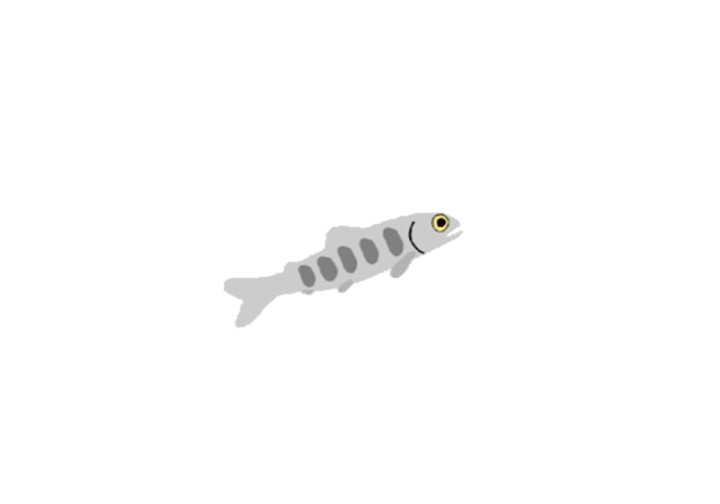

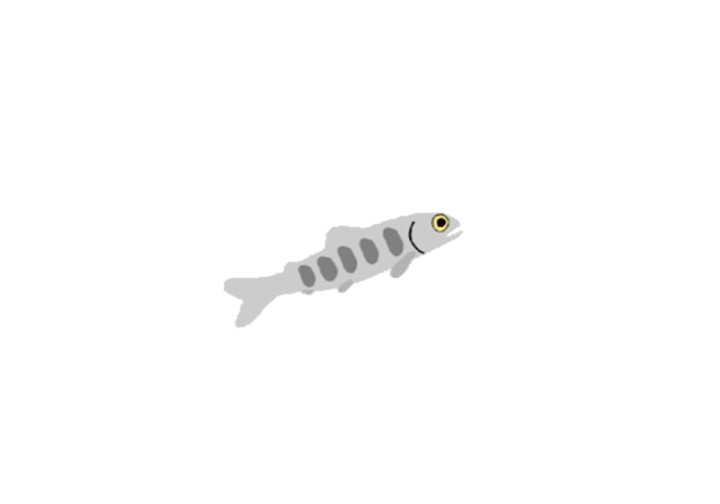

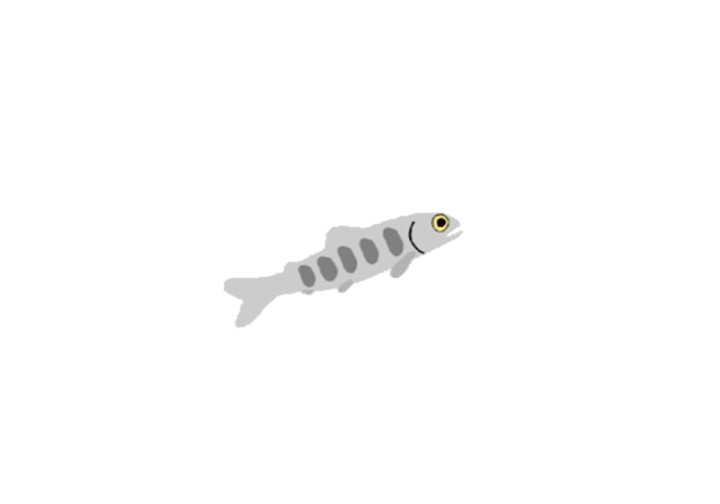

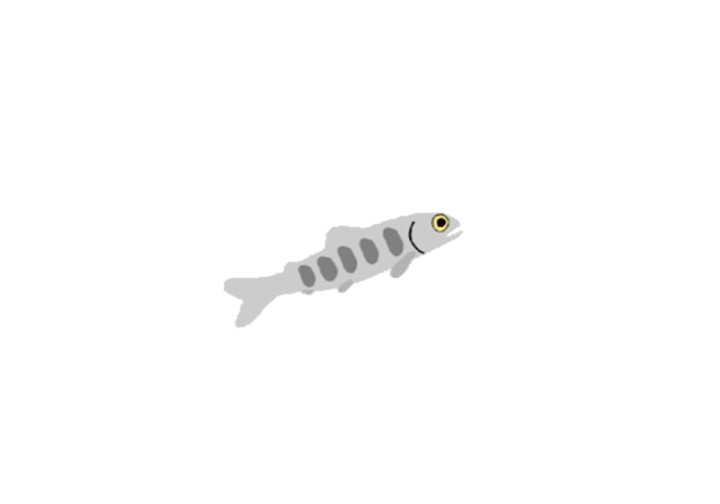

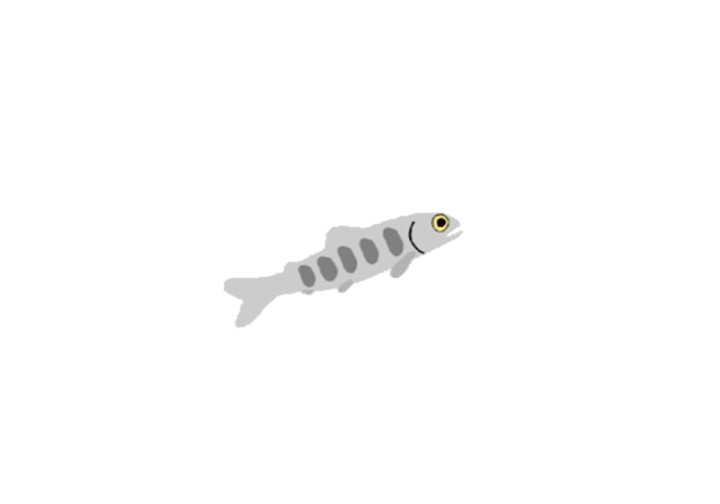

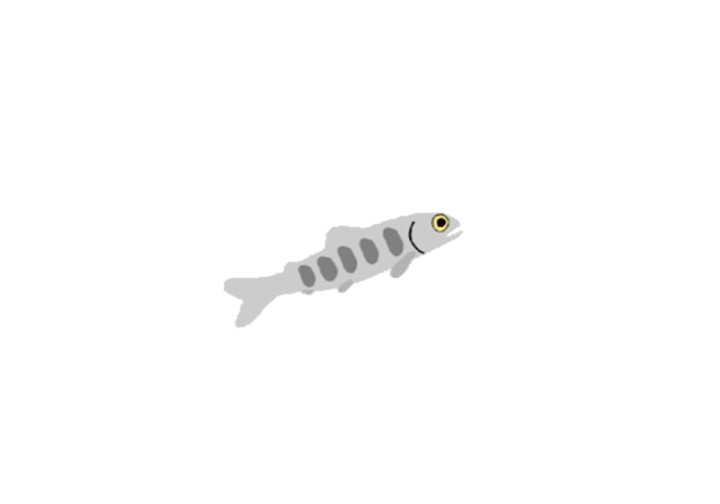

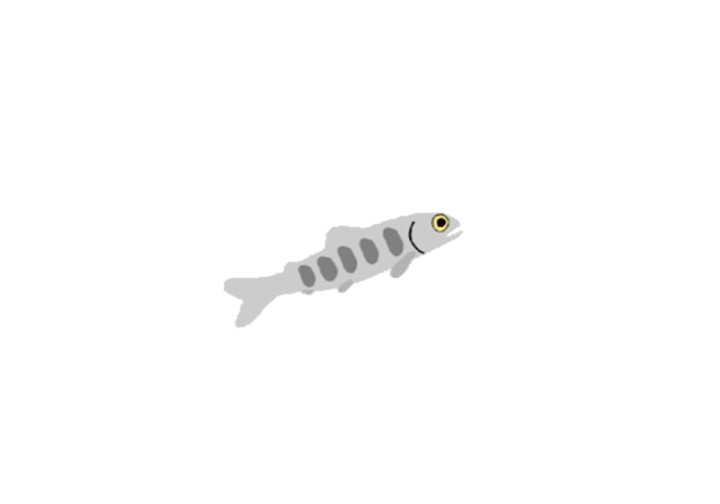

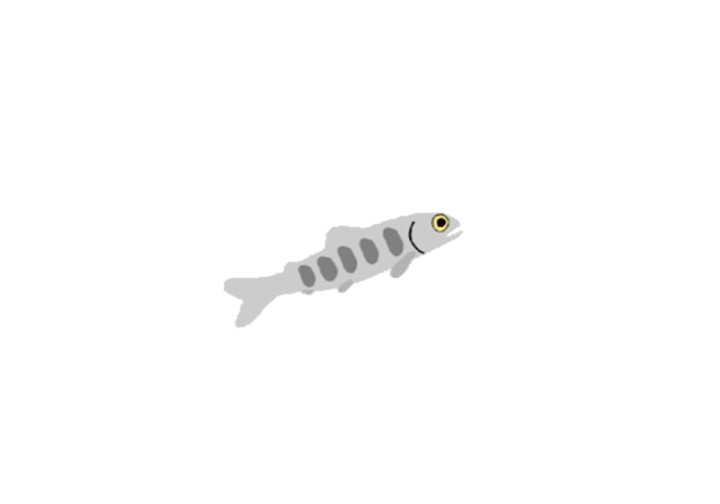

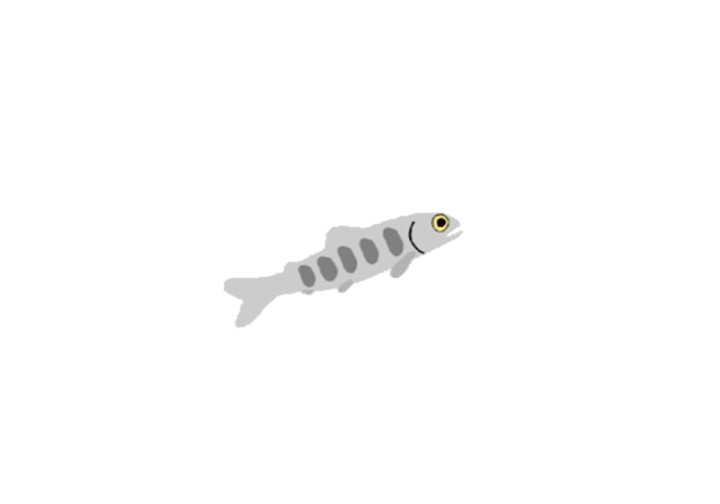

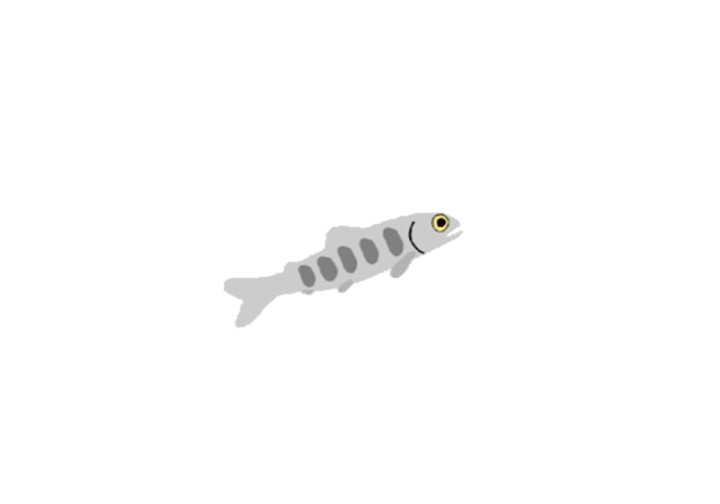

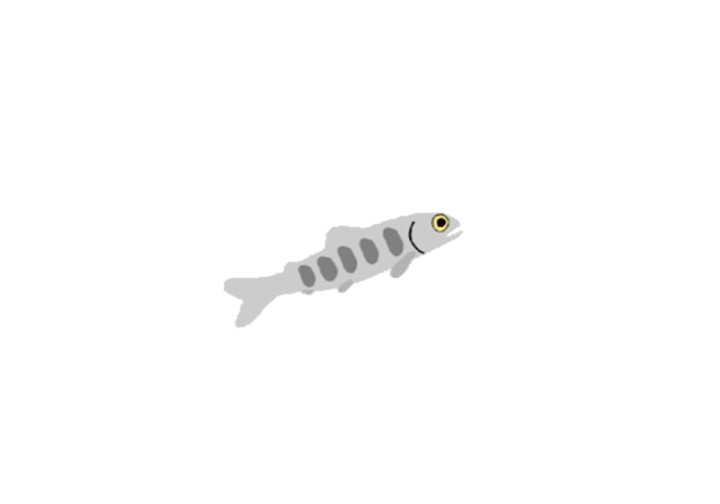

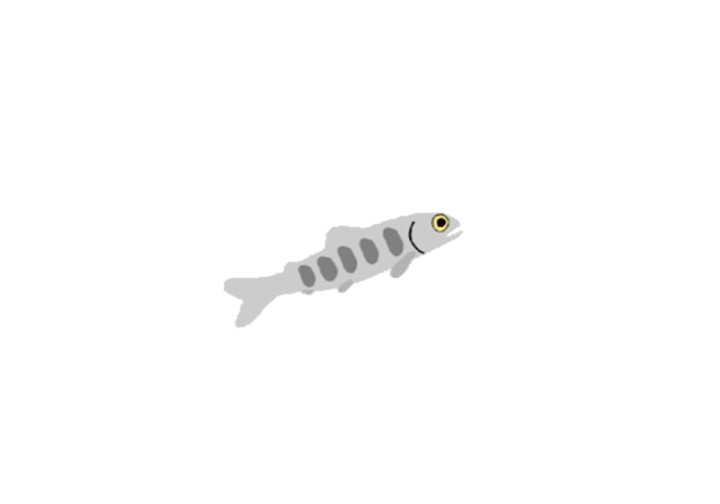

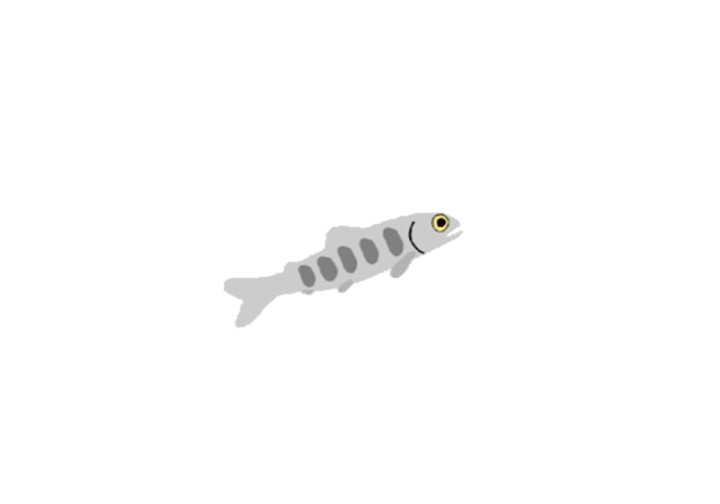

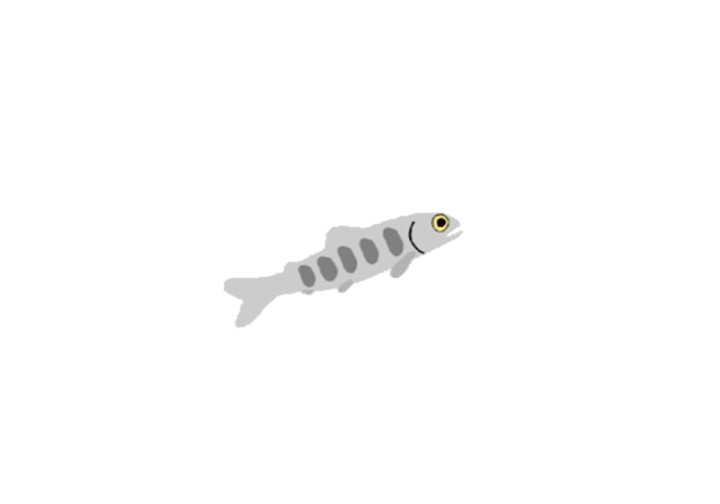

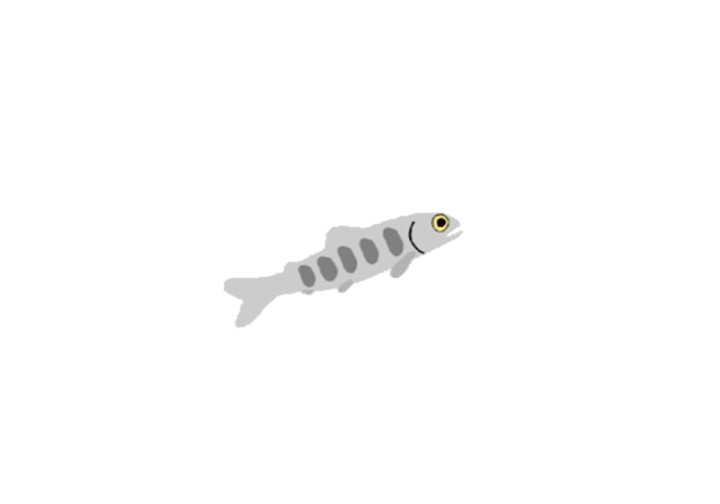

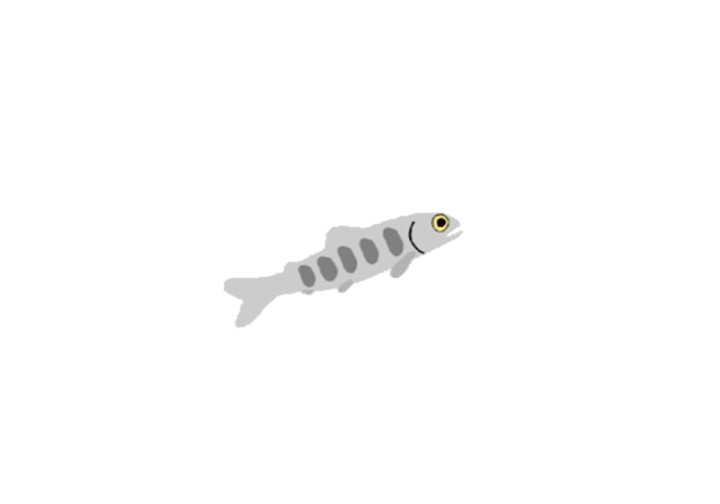

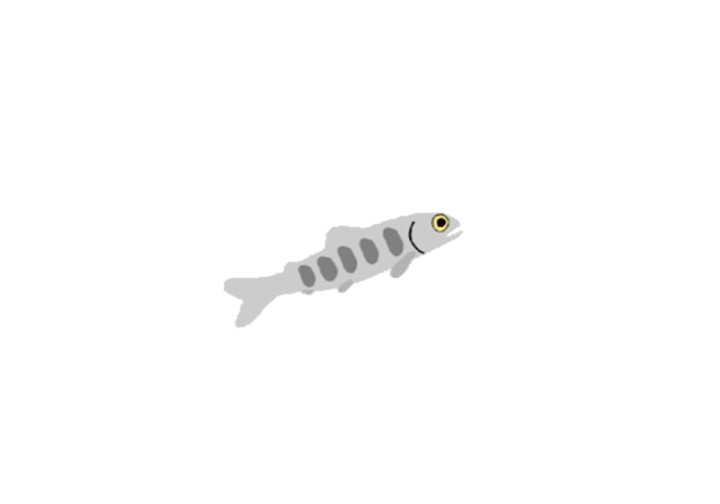

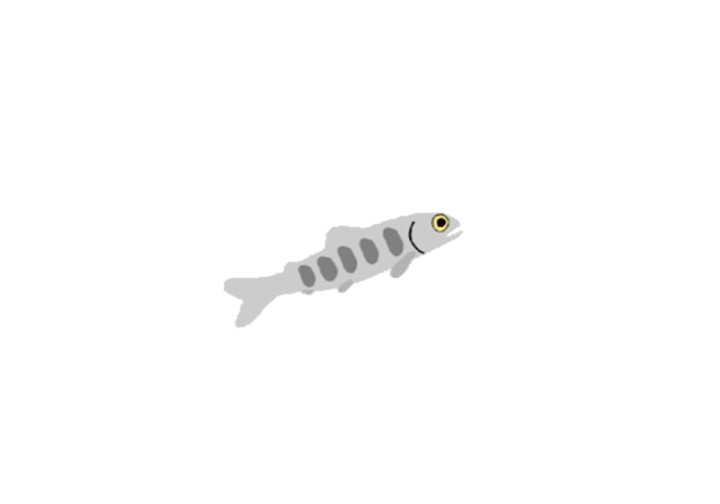

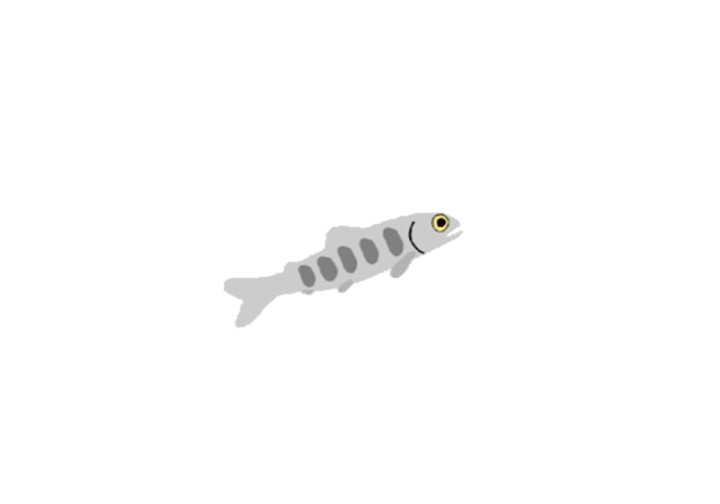

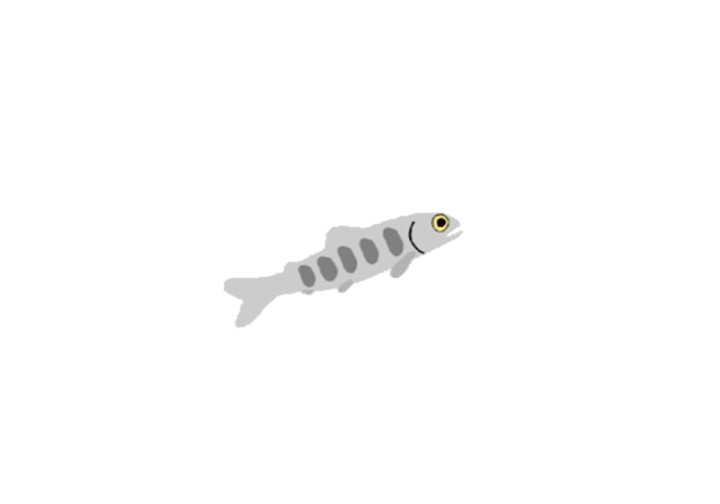

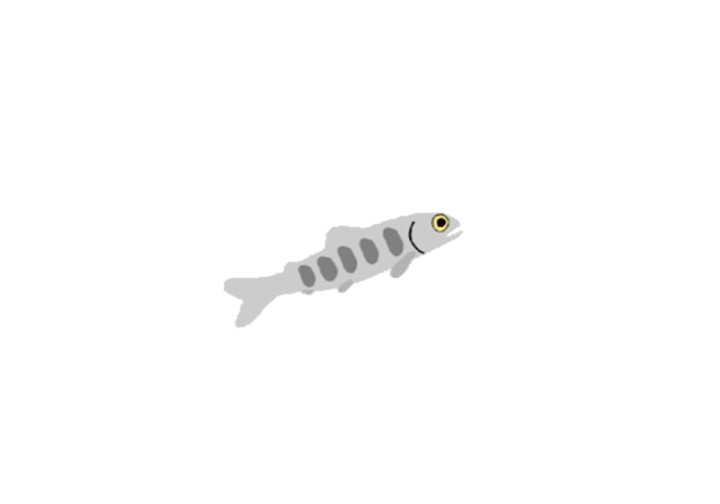

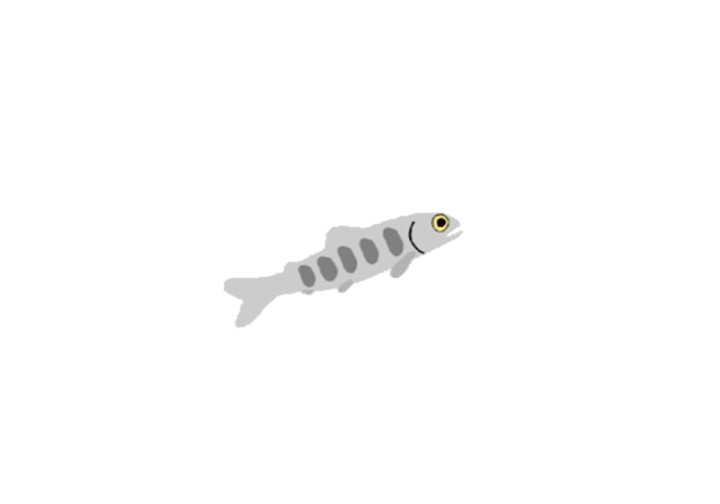

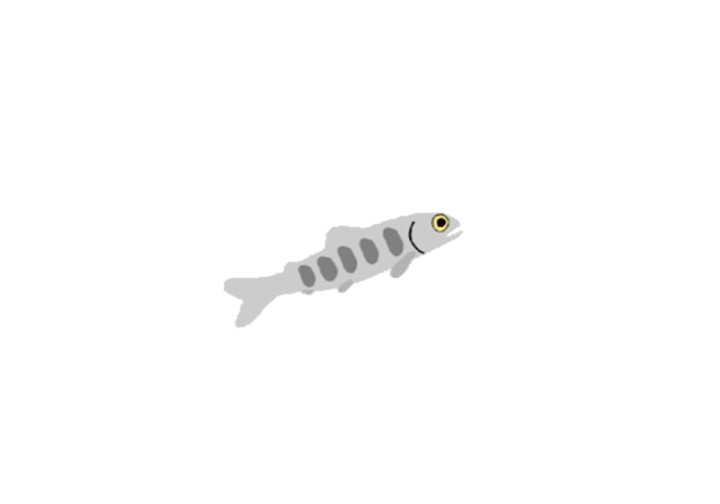

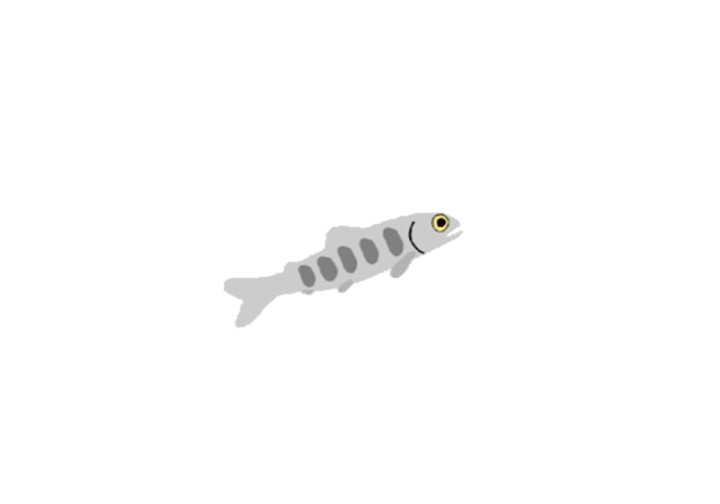


= **High n-3 LC PUFA diet**

= **Low n-3 LC PUFA diet**


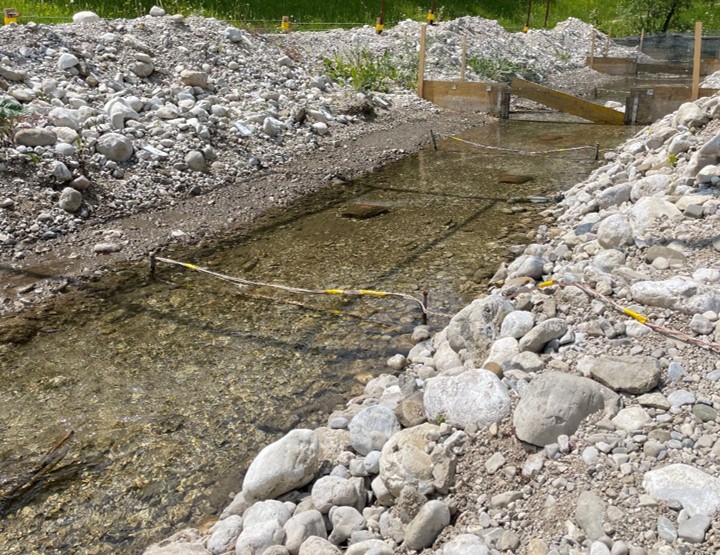


***Figure S3:*** *a) Schematic representation of the structure HyTEC Flume facility designed for the experiment; b) HyTEC Flume facility: the picture shows 1 out of 8 section of the entire mesocosm. Lifted up wooden barrier allowed the moving of a bigger volume of water decreasing the water height.* *Flowering pots worked as shelters.*

***Table S1***: *Summary table of the prey found in brown trout stomach content. Prey are classified at the deepest taxonomic level (i.e. Family) and categorized based on their developmental stage (adult or larvae) and whether they were drift or benthic.*

| ***Order*** | ***Family*** | ***Developmental stage*** | ***Ecological variable*** | ***Abundance*** |
| --- | --- | --- | --- | --- |
| Odonata | Aeshnidae | Adult | Drift | 3 |
| Coleoptera | Dytiscidae | Adult | Drift | 20 |
| Coleoptera | Dytiscidae | Larvae | Benthic | 7 |
| Coleoptera | Elmidae | Adult | Benthic | 1 |
| Coleoptera | Elmidae | Larvae | Benthic | 24 |
| Coleoptera | Gyrinidae | Adult | Benthic | 4 |
| Coleoptera | Gyrinidae | Larvae | Benthic | 1 |
| Coleoptera | Hydrophilidae | Larvae | Benthic | 3 |
| Coleoptera | Noteridae | Adult | Benthic | 2 |
| Coleoptera | NA | Adult | Drift | 5 |
| Crustacea | Gammaridae | Adult | Benthic | 1 |
| Crustacea | Gammaridae | Larvae | Benthic | 5 |
| Diptera | Chironomidae | Larvae | Benthic | 67 |
| Diptera | Culicidae | Adult | Drift | 13 |
| Diptera | Muscidae | Adult | Drift | 21 |
| Diptera | Tabanidae | Adult | Drift | 1 |
| Diptera | Tabanidae | Larvae | Benthic | 1 |
| Diptera | Tipulidae | Larvae | Benthic | 1 |
| Ephemeroptera | Baetidae | Adult | Drift | 230 |
| Ephemeroptera | Baetidae | Larvae | Benthic | 482 |
| Ephemeroptera | Caenidae | Larvae | Benthic | 10 |
| Ephemeroptera | Heptageniidae | Adult | Drift | 6 |
| Ephemeroptera | Heptageniidae | Larvae | Benthic | 53 |
| Ephemeroptera | Leptophlebiidae | Larvae | Benthic | 1 |
| Ephemeroptera | Tricorythidae | Larvae | Benthic | 6 |
| Hemiptera | Gerridae | Adult | Benthic | 2 |
| Hemiptera | Naucoridae | Adult | Benthic | 2 |
| Hemiptera | Notonectidae | Adult | Benthic | 11 |
| Hemiptera | Veelidae | Adult | Benthic | 5 |
| Hymenoptera | Formicidae | Adult | Drift | 61 |
| Mollusca | Lymnaeidae | Adult | Drift | 1 |
| Mollusca | Physidae | Adult | Benthic | 4 |
| Mollusca | Physidae | Larvae | Benthic | 1 |
| Odonata | Aeshnidae | Adult | Drift | 3 |
| Oligochaeta | Lumbriculidae | Adult | Drift | 4 |
| Oligochaeta | Lumbriculidae | Larvae | Benthic | 69 |
| Plecoptera | Leuctridae | Adult | Drift | 112 |
| Plecoptera | Leuctridae | Larvae | Benthic | 217 |
| Plecoptera | Perlodidae | Adult | Drift | 27 |
| Plecoptera | Perlodidae | Larvae | Benthic | 1 |
| Araneae | Pholcidae | Adults | Drift | 1 |
| Tricoptera | Brachycentridae | Larvae | Benthic | 9 |
| Tricoptera | Hydropsychidae | Larvae | Benthic | 33 |
| Tricoptera | Lepidostomatidae | Larvae | Benthic | 42 |
| Tricoptera | Leptoceridae | Larvae | Benthic | 14 |
| Tricoptera | Limnephilidae | Larvae | Benthic | 6 |
| Tricoptera | Philopotamidae | Larvae | Benthic | 7 |
| Tricoptera | Sericostomatidae | Larvae | Benthic | 1 |
| Unidentified terrestrial insects | NA | Adult | Drift | 155 |
| Unidentified terrestrial insects | NA | Larvae | Drift | 1 |

**a)**

**b)**


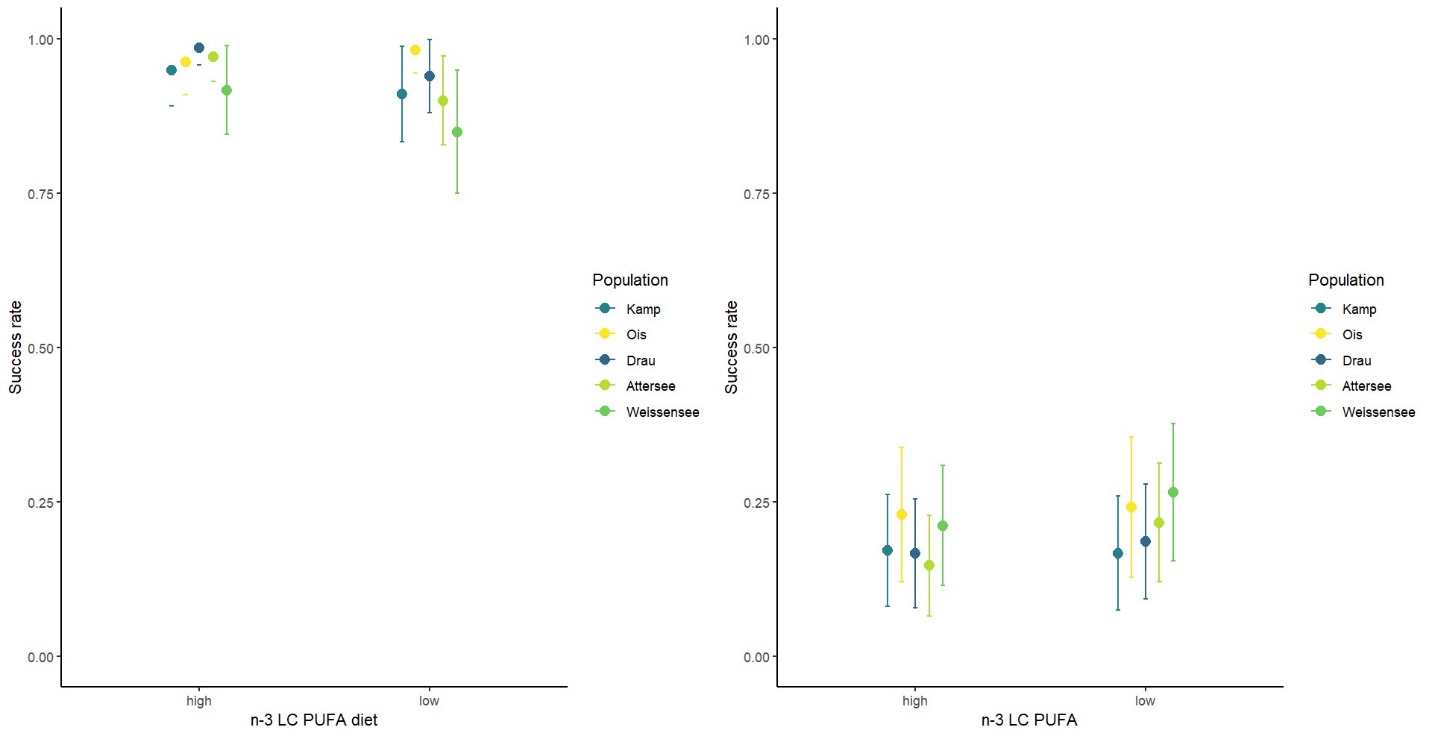


**Figure S4:** Success rate comparison between inhibitory control detour test (ICDT) without (a) and with funnel (b). The scatter plot with error bars shows the success rate (y-axis) from five populations (Kamp, Ois, Drau, Attersee, Weissensee) under high (red) and low (blue) n-3 LC-PUFA dietary treatments. Each population has two points and a vertical line, indicating confidence intervals. Notice that the rate in the first version of the ICDT is far higher than the second. This difference made the model for the ICDT without funnel not converge.


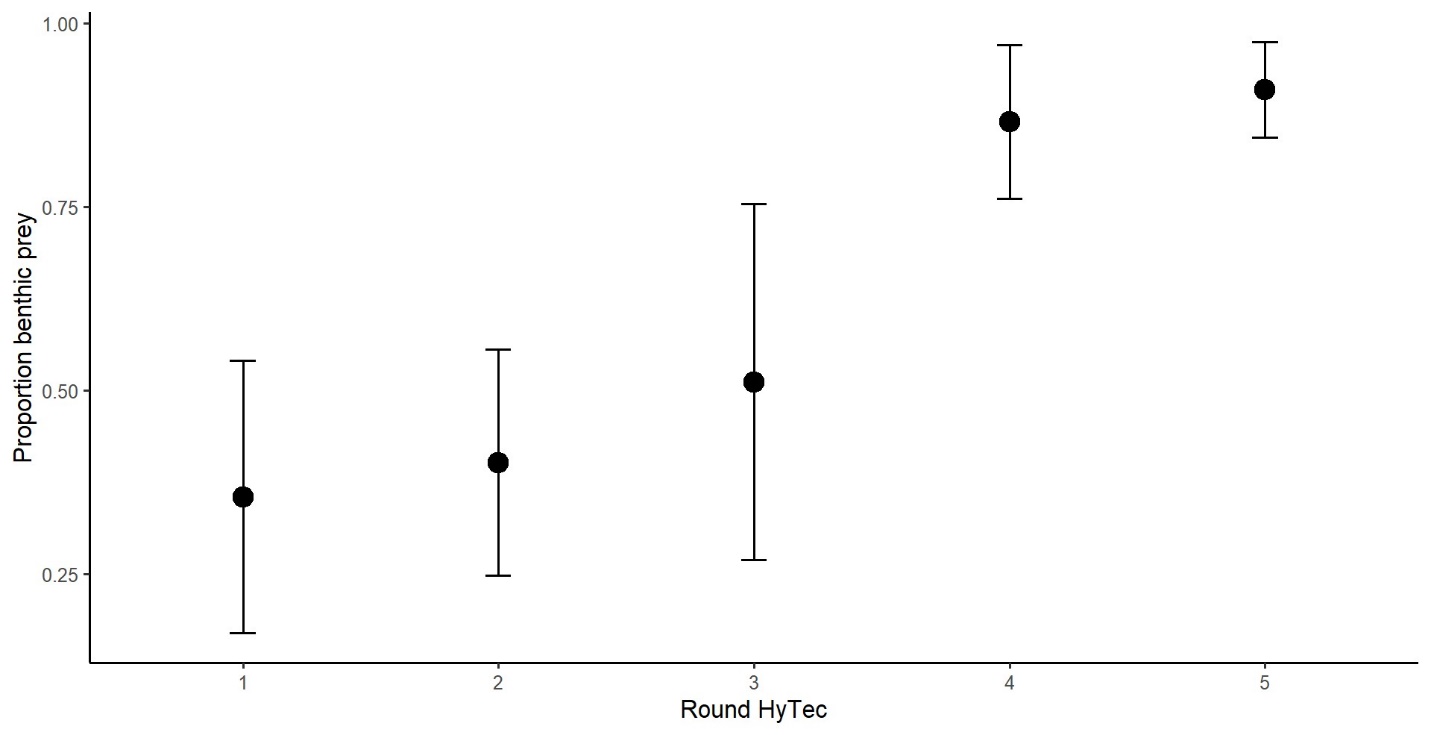


**Figure S5:** Trend of proportion of benthic prey versus drifting prey across the trials found in trout stomach. The scatter plot with error bars shows the proportion of benthic drifting prey (y-axis) from five populations (Kamp, Ois, Drau, Attersee, Weissensee) in the different experimental round in the HyTEC Flume. Each round has one point and a vertical line indicating confidence intervals.
